# Integration of multi-omics approaches for functional characterization of muscle related selective sweep genes in Nanchukmacdon

**DOI:** 10.1038/s41598-021-86683-4

**Published:** 2021-03-30

**Authors:** Devender Arora, Krishnamoorthy Srikanth, Jongin Lee, Daehwan Lee, Nayoung Park, Suyeon Wy, Hyeonji Kim, Jong-Eun Park, Han-Ha Chai, Dajeong Lim, In-Cheol Cho, Jaebum Kim, Woncheoul Park

**Affiliations:** 1 Animal Genomics and Bioinformatics Division, National Institute of Animal Science, RDA, Wanju, 55365 Republic of Korea; 2 Subtropical Livestock Research Institute, National Institute of Animal Science, RDA, Jeju, 63242 Korea; 3 Department of Biomedical Science and Engineering, Konkuk University, Seoul, 05029 Republic of Korea; 4 Department of Animal Science, Cornell University, Ithaca, NY 14853 USA

**Keywords:** Evolutionary biology, Functional genomics, Gene expression, Genome, Genomics, Haplotypes

## Abstract

Pig as a food source serves daily dietary demand to a wide population around the world. Preference of meat depends on various factors with muscle play the central role. In this regards, selective breeding abled us to develop “Nanchukmacdon” a pig breeds with an enhanced variety of meat and high fertility rate. To identify genomic regions under selection we performed whole-genome resequencing, transcriptome, and whole-genome bisulfite sequencing from Nanchukmacdon muscles samples and used published data for three other breeds such as Landrace, Duroc, Jeju native pig and analyzed the functional characterization of candidate genes. In this study, we present a comprehensive approach to identify candidate genes by using multi-omics approaches. We performed two different methods XP-EHH, XP-CLR to identify traces of artificial selection for traits of economic importance. Moreover, RNAseq analysis was done to identify differentially expressed genes in the crossed breed population. Several genes (*UGT8, ZGRF1, NDUFA10, EBF3, ELN, UBE2L6, NCALD, MELK, SERP2, GDPD5, *and* FHL2*) were identified as selective sweep and differentially expressed in muscles related pathways. Furthermore, nucleotide diversity analysis revealed low genetic diversity in Nanchukmacdon for identified genes in comparison to related breeds and whole-genome bisulfite sequencing data shows the critical role of DNA methylation pattern in identified genes that leads to enhanced variety of meat. This work demonstrates a way to identify the molecular signature and lays a foundation for future genomic enabled pig breeding.

## Introduction

Pig is the most studied animal model to date with Mitochondrial DNA (mtDNA) analysis tracked down its origin from Eurasian wild-boar^1^. Among other animals, pig share a deep connection with human civilization and played a critical role in fulfilling the feed demands^2^. Estimates for the consumption of animal food suggest that pork demand will rise to 36% of the overall meat consumption by 2025 and hence require attention to sustain and improve meat quality with its production to secure global food demand^3^.

The Korean peninsula is one of the largest pig consuming country with its Jeju native black pig (JNP) is an indigenous variety of Korean pig with high-quality meat content, redness and nutrition value^4,5^. Superior taste leads to an increase in JNP demand with every passing day but less productivity and fertility made it difficult to sustain the demand^6,7^. Due to huge demand but less productivity intrusion of different breeds leads to diversion of industry focus on alternative economic viable options^8,9^. These imported pig breeds possessed the excellent genetic potential for high production and their growth have been reported to more than 0.5 kg of weight per day but limited with meat quality content^10,11^. This ultimately threatened the indigenous variety of pig with almost reached extinction until the government put serious effort and involved in saving the native pig breed by close monitoring the growth and use.

To address the issue and sustain the demand of JNP in the entire region with food security for the long run, the emphasis has been given to developing a breed with high productivity and meat quality. An in-house breeding program was started to develop a breed with indigenous pig features and have a high fertility rate. Marker-based multiple inter-crosses using a strict selection of breeding pigs (Jeju Native black pig, Duroc, Landrace) accelerated the generation of outstanding progeny containing high meat-quality breed. In the course of continuously close monitoring and breeding program using modern biological methods, Nanchukmacdon a mixed breed was developed which maintained superior characteristics features in generations. A blind test for sensory reflexes to Nanchukmacdon was performed for meat quality and it was voted similar or better values than those for bacon meats. The carcasses of these newly developed black pigs (Nanchukmacdon) showed the significantly higher levels of intramuscular fat deposition (p < 0.05), the redness and the yellowness (p < 0.05) but not in the lightness (p > 0.05) in meat color contents^12^.

Identification of genomic regions undergone positive selection is a potent approach to delineate genes that help in adaptation to environmental factors and responsible for the phenotypic diversity. In the last decade, many GWAS studies have been conducted in this regards and supported by statistical advancement analysis to pin down significant results from the driven data. These approaches already helped in the identification of different genomic regions with selection signals, suggesting the contribution of the region in influencing certain characteristics related to phenotypic or genotypic composition in different breeds. To further extent, genes identified from whole-genome sequencing (WGS) may have evolved to adapt to the conditions but identifying the one expressing and controlling the trait features ideally govern these characteristics and identifying such differentially expressed genes for different trait could help us in identifying markers of interest to develop breeds with such high potential. In this search, RNAseq and whole-genome bisulfite sequencing (WGBS) analysis approaches are the attractive approach for the identification of differentially expressed genes and analysis of methylation role and have been used in various studies regarding such analysis^13–15^.

In this study, we have evaluated the genetic closeness of Nanchukmacdon with the related species and identified the selective sweep genes that allow the enhancement in the characteristics features possessed by the breed. We presented an unbiased approach combining WGS from different breeds of pig, RNAseq data from muscles of closely related species amongst them. We used statistically established methods such as cross-population extended haplotype homozygosity (XP-EHH)^16^ and cross-population composite likelihood ratio (XP-CLR)^17^ statistics to detect selection signatures from closely related breeds and Nucleotide Diversity analysis was performed in Nanchukmacdon using vcftools^18^. Finally, WGBS data analyzed for Nanchukmacdon to observe the methylation pattern in identified genes.

## Results

### Population structure analyses

**Figure 1:**
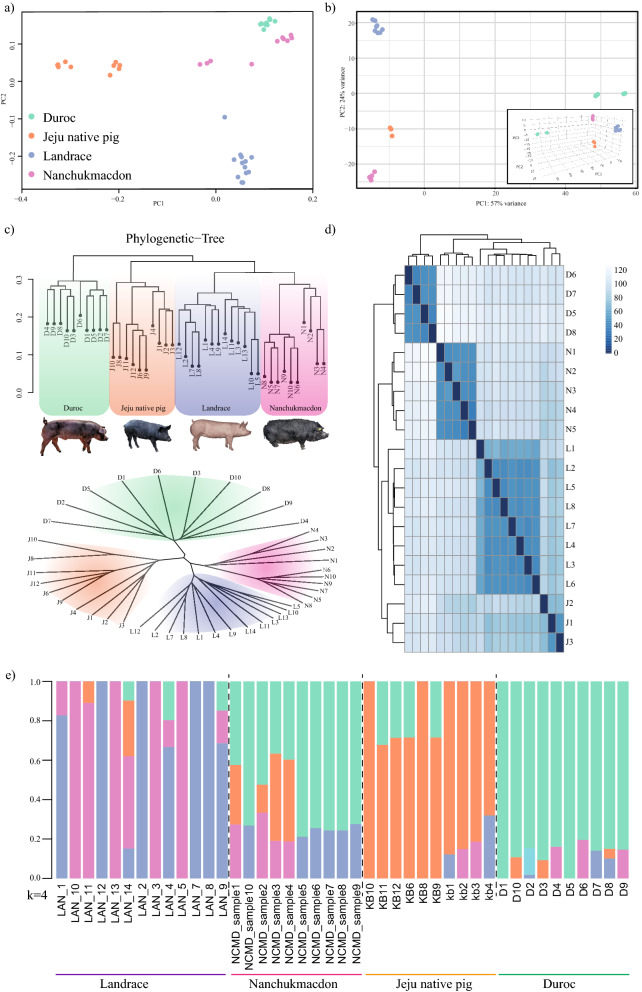
Population structure analyses for all pig individuals. (**a**) First and second principal components from a principal component analysis of all populations WGS data. (**b**) PCA for muscles RNA-seq data distinct the population group. (**c**) Rooted and un-rooted tree representation of related closeness amongst different pig breed. (**d**) Heat-map visualization of common DEGs: column represent DEGs from muscle and row represent assemble method from 20 pig samples. (**e**) Population structure plots for all pig populations at K = 4.

PCA plot analysis describes the separation of species in 2 dimensional view. From the present analysis on 43 resequencing and RNA-seq sample analysis, we observed the clear association of 4 species. Nanchukmacdon is a breed between duroc, Landrace, and JNP and from the plot, it is conclusive that the genetic constituent is closer to duroc and with a certain contribution of JNP (Fig. 1a,b).

### Positive selective signature in Nanchukmacdon population

In total, we have obtained 1154, 1296, and 1666 putative selection regions with p-values < 0.05 in XP-EHH and top 1% score limited to 574, 745, and 675 in XP-CLR putative positively selected genes test statistics in Nanchukmacdon from the three breeds Duroc, JNP, and Landrace respectively (Additional_data 1). Based on the analysis, we further narrow down the obtained results by overlapping XP-EHH and XP-CLR results and limited to 37, 41, and 39 genes in the statistical analysis.

### Identification and analysis of differentially expressed genes (DEGs) in muscle tissue

**Figure 2:**
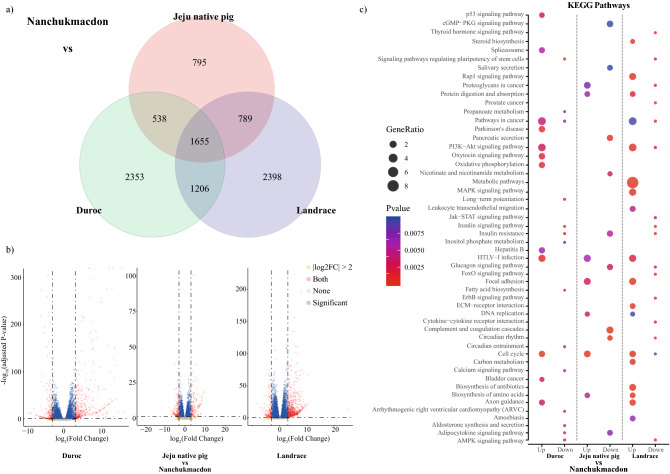
Result of RNAseq analysis of Nanchukmacdon with Duroc, Jeju native pig and Landrace. (**a**) Commonly identified differentially expressed genes. (**b**) Volcano-Plot for distribution of adjusted p-value with log2foldchange for DEGs analysis in muscles against Duroc, Black pig and landrace with Nanchukmacdon respectively and (**c**) KEGG pathway enrichment analysis after functional annotation with p < 0.01. Enriched pathway in Nanchukmacdon from the different breed was performed by dot-plot analysis.

DESeq was used to identify statistically significant differences in gene expression obtained by featurecounts^18^. A cutoff value of fold change ≥ 1 and adjusted FDR correction p-value < 0.05 was selected to obtain DEGs between different breeds NC_JNP_Muscles, NC_DU_Muscles, and NC_LR_Muscles (Additional_data 2). Amongst identified DEGs 1655 were found common in all the breeds (Fig. 2a). The overall relationship between different breed was depicted by Volcano Plot (Fig. 2b).

### Gene ontology and functional profiling studies

**Figure 3:**
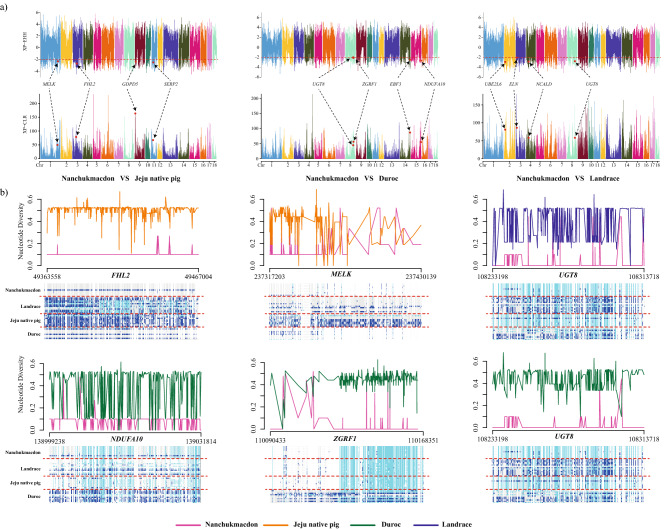
(**a**) Candidate selective sweep analysis for NC. A window size of 10k with the binning size of 10 was chosen and top 1% XP-CLR scores were extracted and a cutoff of -2 with significant p-value ≤ 0.05 were selected for XP-EHH scores. Commonly identified genes were subsequently mapped with differentially expressed genes. Positively expressed DEGs were indicated in red color. Nuclear diversity plot for the identified candidate genes (**b**) represent the nuclear diversity at each point of location and their respective haplotype distribution with genomic position biallelic alleles are shown in sky-blue (homozygous variant) and blue (Heterozygous variant).

Separate analysis was performed for identified positive selective sweep genes identified from XP-EHH and XP-CLR score and DEGs in Nanchukmacdon from Duroc, JNP, and landrace. The functional annotations of genes were categorized into three groups such as molecular function, cellular component, and biological process. Most significant (corrected p-value < 0.05) GO terms such as regulation of synaptic plasticity (GO:0048167), Lipid catabolism (GO:0016042), and post-chaperonin tubulin folding pathway (GO:0007023) were identified in the biological process of XP-EHH. Likewise, in the case of XP-CLR positive regulation of neuron differentiation (GO:0045666), cytoskeleton organization (GO:0007010), and brown fat cell differentiation (GO:0050873) were identified in biological processes. Similarly, molecular and cellular function ontology was also performed and observed the involvement in kainate selective glutamate receptor activity (GO:0015277), neuromuscular junction (GO:0031594), ruffle membrane (GO:0032587) are some. Similarly, Gene Ontology (GO) of DEGs reveals that the significantly expressed genes related protein ubiquitination involved in ubiquitin-dependent protein catabolic process (GO:0042787), cytoskeleton-dependent intracellular transport (GO:0030705), angiogenesis, extracellular matrix organization, negative regulation of inflammatory response are some in biological processes. Likewise, we have seen critical involvement of extracellular exomes, membrane, proteinaceous extracellular matrix enrichment in cellular compartment (Supplementary Fig S1a). KEGG pathway analysis reveals the involvement of major pathways varies from Metabolic pathway, Fatty acid biosynthesis, ErbB signalling pathway, Adipocytokine signalling pathway, Calcium signalling pathway and Oxidative phosphorylation are some (Fig. 2c, Supplementary Fig. S2). Manhattan plot for muscles WGS data analysis was generated for NC with DU, JNP, and LR by XP-EHH and XP-CLR score and annotated with commonly identified differentially expressed genes with CMplot^19^. XP-EHH and XP-CLR score was plotted against the genomic position with the autosomal chromosomes in different colors (Fig. 3a).

### Validation of identified genes

**Figure 4:**
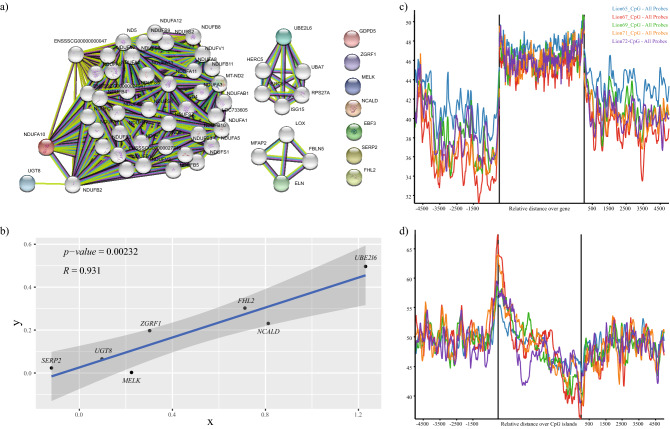
Related protein association and validation was performed. (**a**) All the identified genes were visualized using string database. (**b**) RT-PCR result for randomly selected genes showing high correlation and significant p-value or r = 0.931 and p-value = 0.00232. (**c**,**d**) Methylation pattern where a relative degree of gene stabilization can be seen and (**d**) sharply decreasing at TSS region of CpG island and stabilizing afterwards.

All the identified genes were incorporated in the string database for protein–protein interaction analysis and it was observed that they share different modules of networks in governing important characteristics features (Fig. 4a)^20^.

The muscle tissue from Nanchukmacdon was collected and used for expression analysis for seven randomly selected genes. The transcripts selected for validation are *SERP2, UGT8, MELK, ZGRF1, FHL2, NCALD, UBE2L6* as mentioned in Additional_table 3. The RT-PCR experiment for selected samples was executed with three replicates for each sample. *GAPDH* and Beta-actin were used as an endogenous control for normalizing the quantification cycle (Cq) value. The contrast is depicted in (Fig. 4b). Results indicate that the expression of selected genes to control is significantly correlated with high confidence p-value = 0.00232 and R = 0.931.

WGBS data analysis was performed for Nanchukmacdon to analyze the methylation in identified genes. As expected from the observed pattern, DNA methylation level sharply decreased near 5 kb upstream region of TSSs and dropped to the lowest outside the region (Fig. 4c), methylation level remains stable after promoter region contributing to structural stability and regulation of gene expression. CpG Island was less expressed inside than outside of 5 kb CpG Island (Fig. 4d)^21^. Individual methylation pattern for all the identified genes confirms the pattern of no methylation corresponding with the distribution of gene promoters, usually prone to transcription (Supplementary Fig. S3).

## Discussion

Genomic selection has been the main tool supported by statistical analysis in genetic improvement of economically important traits^22^. Selective signature ideally helps in stabilizing traits that make a breed unique with its features. Studying these traits allows a better understanding of breed and help in developing enhanced breed with high economic values. In this regards, Nanchukmacdon, a mixed breed was developed that exhibits exceptional meat quality with a high fertility rate and performed selective sweep analysis to understand the genes that triggered the meat characteristics. WGS data has been used for various GWAS related studies^23–26^, Incorporation of RNAseq analysis approach to WGS data provide us with a more detailed and better understanding to the identified genes and how they behave in the present system which makes specific trait different than the parental generations. Recently, various studies have been published reporting selective genes in different breeds over a period of time with main focus on identifying selective signature associated with the breeds and how these genes expressing in the system^27–29^. As meat quality is accessed in terms of the carcass, feed conversion efficacy, color, taste, juiciness and majorly consist of 75% of the muscles tissue^30^. In this regards, to identify selective sweep genes in closely related breeds we used statistically established methods such as cross-population extended haplotype homozygosity (XP-EHH)^16^ and cross population composite likelihood ratio (XP-CLR)^17^ statistics in order to detect selection signatures from closely related breeds; two approaches were used as each has its own advantages. XP-EHH compares haplotype lengths of populations to detect selective sweeps when the allele has approached or achieved fixation in one population but remains polymorphic in the other population. XP-CLR is a statistic based on allele frequency differentiation across populations providing an advantage to detect older signals and selection on standing variation. Subsequently, we integrated RNAseq analysis data of muscles to identify the muscles associated genes in the mixed breed with their closed associated pig varieties.

After identifying common genes amongst different varieties exhibiting positive selective signature identified using XP-EHH and XPCLR statistical test, identification of common genes expressing in muscles tissue of Nanchukmacdon limited the total number of genes amongst different breeds to 11 genes with 4 each in JNP, DU, and LR respectively. Amongst *UGT8* was identified in DU and LR. Here, JNP 4 (*MELK, SERP2, GDPD5, FHL2*) genes, DU (*UGT8, ZGRF1, NDUFA10, EBF3*) genes and in LR (*UGT8, ELN, UBE2L6, NCALD*) genes were identified [Table 1]. Similarly, identification of negatively expressed genes in Nanchukmacdon has been performed and common genes amongst JNP, DU, and LR with the cutoff parameter for log2fold change > 1 were identified to be limited with 13 genes, Additional_table 1.

**Table 1:** Commonly identified selective signature genes with top 1% of XP-CLR and -2 cutoff for XP-EHH score with log2fold change of < − 1 and FDR of 0.05 against JNP, Duroc and Landrace.

Compare	Chr	Gene symbol	ENS_ID	XP-CLR	XP-EHH	log2FC	FDR
Nanchukmacdon vs. Jeju Native Pig	1	MELK	ENSSSCG00000005344	50.31517901	− 2.312935631	− 2.18	0.000124034
	11	SERP2	ENSSSCG00000040405	67.168271	− 2.485311147	− 2.15	0.008392278
	9	GDPD5	ENSSSCG00000014853	163.2458645	− 2.364323658	− 1.63	0.00000212
	3	FHL2	ENSSSCG00000008147	78.45288699	− 2.696124665	− 1	0.000516892
Nanchukmacdon vs. Duroc	8	UGT8	ENSSSCG00000031904	44.70694034	− 2.024535069	− 3.64	0.003195103
	8	ZGRF1	ENSSSCG00000009120	56.23853217	− 2.164609468	− 1.52	0.003773682
	15	NDUFA10	ENSSSCG00000016349	55.03739176	− 2.714584707	− 1.24	0.00000000000000181
	14	EBF3	ENSSSCG00000010757	86.87865636	− 2.505302829	− 1.09	0.0000000000063
Nanchukmacdon vs. Landrace	8	UGT8	ENSSSCG00000031904	57.77182778	− 2.738613817	− 2.34	0.000178915
	3	ELN	ENSSSCG00000025858	85.97446047	− 2.080438144	− 1.94	0.0000000000000772
	4	NCALD	ENSSSCG00000006059	58.25052636	− 2.861748569	− 1.05	0.0000000314
	2	UBE2L6	ENSSSCG00000023379	80.85200729	− 2.939038774	− 1.14	0.000000515

Our findings on the genetic relationship amongst different breeds agree with the previously reported studies on different pig breeds using WGS data^31^, signifying the grouping of Nanchukmacdon with Duroc, Jeju native pig, and Landrace (Fig. 1a–c) with visualization of DEGs were performed using heatmap analysis (Fig. 1d). The individuals from the Nanchukmacdon, landrace, duroc and JNP population were grouped according to their origin as identified by PCA (Fig. 1a,b). Our results indicate that Nanchukmacdon is phylogenetically closer to landrace (Fig. 1c). To estimate individual ancestry, admixture proportions were assessed without defined population information using ADMIXTURE with The individual population was grouped into separate clusters at K = 4 with the lowest cross validation error (Fig. 1e and Supplementary Fig. S4) and these results were also confirmed from unrooted tree (Fig. 1c). In selection signature analysis, we further performed a comparative analysis with closely related breeds. Our study reveals a series of well-known and novel genes reported in muscles related to biological processes and their high expression pattern after getting positively selected in the mixed breed. RNAseq methodology allowed us to further understand the changes held in selective sweep genes during the evolution. We have seen major expression alteration in mineral content or related pathways by the different expression pattern of selective sweep genes specifically muscles related (*FHL2, EBF3*), calcium ion channel route (*NCALD, UBE2l6*), metabolism, and fatty acid metabolism pathways (*UGT8, GDPD5, MELK*) when it compares to their closely related breeds. The genes under selection were further investigated using Nucleotide diversity approach for the selective sweep genes. Low nucleotide diversity signifies the stabilization and activation of genes under specific circumstances^32,33^ and in Nanchukmacdon with their respective paired breed and identified the low diversity reported over the genomic coordinates and haplotype diversity in comparison to all other breeds (Fig. 3b, Supplementary Figs. S5 and S6).

Amongst identified genes, UDP Glycosyltransferase 8 (*UGT8*) was found to be highly expressed in Nanchukmacdon w.r.t. Duroc and Landrace variety of pig. Although there is limited understanding of *UGT8* role with meat quality previous studies relates its involvement in either lipid metabolism, sphingolipid metabolism, or in metabolic pathways and majorly reported as over-expressed and in a study by Meech et al. suggested the role in bile acid as Galactosidation of Bile Acids^34^. Bile acid plays a crucial role in controlling lipid and glucose metabolism and play central role in energy metabolism which directly have direct involvement with fatty acid pathway^35^. Since various finding suggests *UGT8* involvement in modulating bile acid signaling our results concludes a strong relation of *UGT8* expression in enhancing meat quality specifically to fat-related pathways. DAVID annotation^36^ also confirmed the functional association of *MELK* in various biological processes. *MELK* a kinase family protein found to be positively expressed in NC_JNP analysis is located in various part of the cell such nucleus, cytoplasm, plasma membrane and cell cortex involved in various processes such intrinsic apoptotic signaling pathway in response to oxidative stress, peptidyl-tyrosine phosphorylation, intracellular signal transduction, positive regulation of the apoptotic process, protein auto-phosphorylation, neural precursor cell proliferation. Interestingly, earlier studies reported the absence of *MELK* expression in muscles in a different animal model^37^ and in contrast, we have reported a log fold change of -2.18 change w.r.t JNP. *MELK* expression was strongly correlated with genes that play role in mitosis (M) phase of the cell cycle^38^ and reported in selective sweep study fat rump of sheep^39^. Whereas, *FHL-2* (The-four-and-a-half-lim) a selective sweep gene identified after XP-EHH and XP-CLR studies and differentially expressed in Nanchukmacdon and reported to have a role in the assembly of extracellular membrane and regulator of fatty acid metabolism and control energy homeostasis in pig^40^. Predicted functional partner from string database also confirmed a close association with epidermal growth factor receptor that could affect the morphology of the muscles^41^. Expression of muscles related genes significantly influenced by the expression pattern of *FHL2* and found to control various genes such as *MyoD1*, *MyH3* and *MyoG* that play a central role in the development of muscles. Similarly, *NCALD* positively selected gene from NC_LR presents in intracellular cellular component and observed for their role in calcium mediated signaling. It is a calcium sensor which directly interacts regulates actin and clathrin. Similarly, *UBE2L6* was reported in molecular functions related to ATP-binding, energy metabolism and nucleotide-binding. It is involved in ubiquitination of multiple substrates^42,43^ and direct involvement in obese related pathways^44^. *GDPD5* or glycerophosphodiester phosphodiesterase 2 (*GDE2*) was identified as positively selective sweep genes whose functional annotation and string database information for protein–protein interaction analysis resulted in the limited lead for direct involvement in any pathway or function but suggested a close association with *ACSL3* gene which play a central role in fatty acid oxidation^45^. *ELN* identified as the positively expressed selective gene found in the extracellular matrix that provides structural support, biochemical or biomechanical cues for cells or tissues by structure lying external to one or more cells^46,47^. *ZGRF1* is not well characterized and function is not known in *Sscrofa* but the gene is reported to be associated with translation, transcription, nonsense-mediated mRNA decay, RNA decay, miRNA processing, RISC assembly, and pre-mRNA splicing. *EBF3*: Positive selection of *EBF3* leads to an up-regulation of myogenic regulatory factors including MyoD and Myf5 required for muscles development^48^. The function of *Ebf3* outside of the neuronal system, however, has limited understanding. *EBF3* play role in muscles specific transcription and have critical role in relaxation by directly regulating the expression of a Ca^2+^ pump^49^. *SERP2* (stress associated endoplasmic reticulum protein family member 2), a positively selected differentially expressed gene with limited understanding of any direct role in muscles related pathway or any process in pig is reported to be differentially expressed in splay leg piglets^50^.

## Conclusion

It is a well-established fact that artificial selection has greatly shaped pig genomic variability during the process of domestication. The variation developed during the event helps the local industry to proliferate and fulfil the local meat demands efficiently. Our primary objective of this study was to identify selective sweep genes that were expressed in muscles and we were able to limit 13 potent genes with their important role in the muscles building process. GO analysis showed various pathways vary from regulation of synaptic plasticity, clathrin-dependent endocytosis, and positive regulation of neuron differentiation were significantly enriched. Similarly, KEGG pathway showed that Metabolic pathways, Calcium signaling pathway, and Endocytosis were significantly enriched in selective sweep genes. These results provide a better understanding of the role of identified genes in regulating muscles related pathways and further information to genomic evolution and selective mechanism which could help develop an enhanced breed with high muscles content.

## Methods

**Figure 5:**
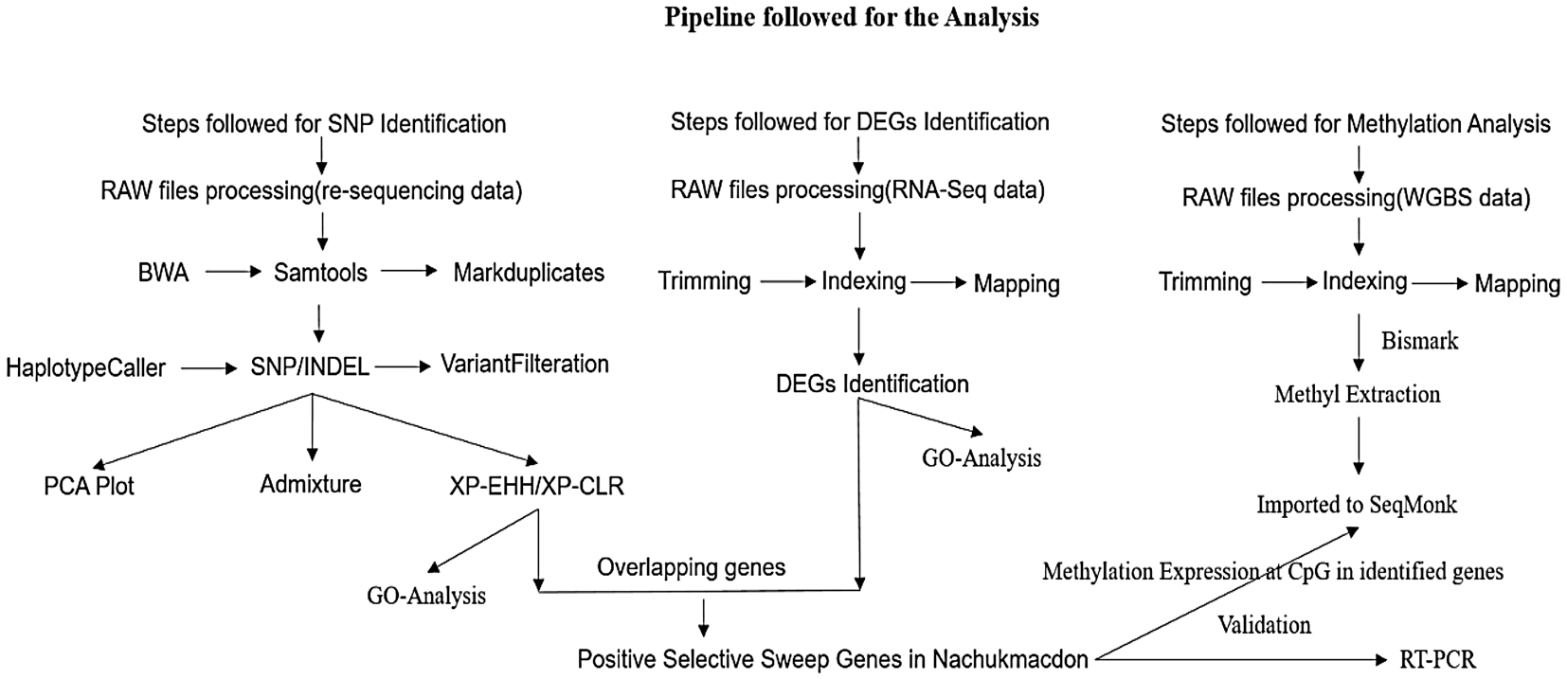
Overview of the pipeline followed to identify selective sweep genes.

To identify selective sweep genes in Nanchukmacdon we have performed multiple analysis and a flowchart has been developed for better understanding of the work (Fig. 5).

### Sampling and data collection

This study aimed to find selection signatures of selective sweep genes using WGS data and integrating RNAseq analysis approaches in Nanchukmacdon, and other pig breeds (JNP, Landrace, and Duroc) to identify differentially expressed selective sweep genes and their role in the biological process. Samples were taken from healthy Male pigs belong to the same farm in Jeju Island with an average age of 2 years for (N = 1–6), and 3 years for (N = 7–10) (Additional_Table 4). All the experimental procedures were verified and approved by the National Institute of Animal Science, and carried out in compliance with the ARRIVE guidelines^51^. Whole-genome re-sequencing was performed from the blood sample taken from post-harvest Nanchukmacdon (N = 10), RNAseq data was generated for Nanchukmacdon (N = 5) pair-end data after isolation of muscle tissue using TRIzol method following the manufacturer guideline and reported earlier^15^. Similarly, gDNA from Nanchukmacdon muscles was subjected to bisulfite conversion using the fragment size (250 bp ± 25 bp), WGBS was performed with MethylMiner Methylated DNA Enrichment kit, and then a sequencing library was constructed using the Illumina Paired-end sequencing on an Illumina, NovaSeq, 150bpX2. Whole genome bisulfite sequencing (N = 5) was performed from the taken sample. The sequencing library was constructed using Illumina NovaSeq RNA sample preparation kit (Illumina, San Diego, CA, USA). Resequencing data for duroc [N = 10], landrace [N = 13], and JNP [N = 10] were retrieved from NCBI and RNAseq data for duroc [N = 4], landrace [N = 8], were retrieved from NCBI and JNP data [N = 3] was collected from the author^52^.

### Sequence mapping and SNP calling

Using Burrows-wheeler aligner tool^53^, depth and genome coordinates were identified after aligning to (*Sus sucrofa 11.1*) reference genome downloaded from NCBI at the default setting. BAM file further cleaned using SAMTOOLS^54^ for low-quality mapping reads and specify permissive quality cutoffs [flag-sat –bS and –bF 4]. The pipeline employs the Genome Analysis Toolkit 4.0 (GATK) to perform variant calling and is based on the best practices for variant discovery analysis outlined by the Broad Institute^55,56^. We used open-source software packages of Picard tools (http://broadinstitute.github.io/picard). Picard tool was used to filter potential PCR duplicates. SAMtools was used to create index files for reference and bam files. Genome analysis toolkit 4.1.4.0 performed local realignment of reads to correct misalignments due to the presence of indels. Further, the HaplotypeCaller, CombineGVCF and “SelectVariant” argument of GATK was used for identifying candidate SNPs^57,58^.

To filter variants and avoid possible false positives, the “VariantFiltration” argument of GATK was adopted with the following options: SNPs with MQ (mapping quality) > 40.0, MQRankSum < − 12.5, ReadPosRankSum < − 8.0 and quality depth (unfiltered depth of non-reference samples; low scores are indicative of false positives and artifacts) < 2.0 were filtered^59^. BEAGLE version 4.1^60^ was used to infer the haplotype phase and impute missing alleles for the entire set of swine populations simultaneously. After all the filtering processes, a total of ~ 26 million SNPs were retained and used for further analysis.

### Phylogenetic tree, admixture and principal component analysis

To accurately describe the population of the crossbred pig, we used SNP data from different breeds and employed SNPRelate R package to perform principal component analysis^61^. Subsequently, Newick file was prepared and viewed in ggtree^62,63^. Results were further analyzed for the distribution of breeds in different coordinates. Similarly, the input file was implemented using unsupervised based clustering method by a program ADMIXTURE to estimate the breed composition of individual animals^64^. The analysis was run with K (number of breeds ‘4’) ranging from 2 to 4 to depict the genetic background of Nanchukmacdon and graphical display of the output was performed in R.

### Detection of genomic regions with putative signals of selection

Using the whole SNP sets defined from NC, DU, LR and JNP, the method cross-population extended haplotype homozygosity (XP-EHH)^16^ and cross-population composite likelihood ratio method (XP-CLR)^17^ was used to detect genome-wide selective sweep regions. XP-EHH assesses haplotype differences between two populations and is designed to detect alleles that have increased in frequency to the point of fixation or near fixation in one of the two populations being compared^65^. Whereas, XP-CLR is based on the linked allele frequency difference between two populations and is a unidirectional method to find the pattern with regional allelic frequency difference in-between population^66^.

Developments in RNA-seq technology enable more comprehensive investigation of the transcriptome for gene expression studies^67^. The statistical analysis is also critical in transcriptomic studies using RNA-seq; specifically, for the normalization of quantitative measurements of expression^68,69^ and detection of DEGs^70^.

The PE reads were checked for the quality assessment using FastQC^56^ and removed low quality reads by Trimmomatic^71^ using parameters leading:3 trailing:3 slidingwindow:4:15 headcrop:13 minlen:36 before proceeding sequence alignment. All quality-filtered PE reads were aligned to *Sscrofa* genome (*Sscrofa11.1*) from the University of California Santa Cruz (UCSC)^72^ using Hisat2^73^ and reads were counted using FeatureCount ^18^. DESeq2 was used to identify differentially expressed genes^74^.

### Gene ontology analysis detection and annotation of candidate genes

Lists of differentially expressed genes with FDR < 0.05 in Nanchukmacdon w.r.t. Duroc, JNP, and Landrace were compiled and submitted to DAVID v6.8 server^36^ for functional annotation and enrichment analysis. For each list, enriched Gene Ontology (GO)^75^ Biological Processes, Molecular functions and Cellular Compartments. These terms were then clustered semantically using the ReviGO server^76^. Enriched functions throughout the whole transcriptome of Nanchukmacdon with elevated GO-term function and the clustered lower-level GO-terms. The letter corresponds to letters found in the treemap for Biological process, Molecular function and Cellular compartment (Additional_data 3, Additional_table 2, Supplementary Fig. S1b). The functional annotation of commonly identified selective sweep genes with differentially expressed genes was performed with the Database for Annotation, Visualization and Integrated Discovery (DAVID) and Kyoto Encyclopedia of Genes and Genomes (KEGG). The genomic coordinates of the regions with high XP-EHH and XP-LCR score for 10k window with 10k bin size were used as input data and used R package BiomaRt^77,78^ to fetch the coordinates against *sscrofa11.1* database for getting the gene_id information of the respective regions with highly significant results^78^. Subsequently, the Database for Annotation, Visualization, and Integrated Discovery (DAVID) (https://david.ncifcrf.gov/^36,79^ was used for Kyoto Encyclopedia of Genes and Genomes (KEGG) pathway^80^ and Gene Ontology (GO)^75^ enrichment analyses. The GO terms and KEGG pathways with corrected p-value < 0.05 were considered significant. REVIGO^76^ and Clusterprofiler R package^81^ were used for summarizing the GO terms.

### WGBS data analysis

The analysis for WGBS data was performed using reproducible genomics analysis pipeline PiGx-bsseq to understand methylation patterns in identified genes^82^. Where sequence was initially performed for a quality check using trim_galore^83^ and alignment were subjected to the filtration of duplicate reads with sam_blaster and sorted using SAMtools^54^ afterwards mapped to the reference genome of *sscrofa11.1* using Bismark^84^. Bismark methyl extractor was performed to measure the methylation in CpG context.

### Validation

WGBS was performed for Nanchukmacdon muscles tissue to see methylation pattern in the identified genes and gene wise methylation visualization was performed to observe methylation level at CpG island by importing the CpG methylation file extracted from bismark methylation extract into SeqMonk visualization tool. The coordinates for identified genes were fetched from Ensembl and each gene was visualized for methylation and found correlating results (Supplementary Fig. S3).

Trizol method (Invitrogen, UK) was used for the total RNA isolation. Qubit fluorometer (Invitrogen, UK), NanoDrop (Thermo Scientific, USA) and Bioanalyzer (Agilent, UK) were used for analyzing the quality of the isolated total RNA. High Capacity cDNA Reverse Transcription Kit (Applied Biosystems™, 4368814) was used for synthesizing the cDNA and RT-PCR was performed by SYBR Green Realtime PCR Master Mix (TOYOBO, QPK-201T).

### Ethics approval and consent to participate

In this study, N refers to number of animals and All the experimental procedures were verified and approved by the Ethics committee of National Institute of Animal with ethical approval no: NIAS20181295.

## Supplementary Information

Supplementary Information 1.

Supplementary Information 2.

Supplementary Information 3.

Supplementary Information 4.

Supplementary Information 5.

Supplementary Information 6.

Supplementary Information 7.

Supplementary Information 8.

Supplementary Information 9.

Supplementary Information 10.

Supplementary Information 11.

Supplementary Information 12.

Supplementary Information 13.

Supplementary Information 14.

Supplementary Information 15.

## Data Availability

The re-sequencing and RNA sequencing was performed on an Illumina Novaseq- sequencer. The raw reads are available for download from sequence read archive (SRA), NCBI under the accession number PRJNA670579. A total of 33 resequencing data and 15 RNAseq data of 3 pigs breed Duroc, JNP and Landrace were retrieved from NCBI with accession number PRJNA260763 and RNAseq data were retrieved from PRJNA392949, Landrace PRJNA488993, and JNP data from Ghosh et al.^52^.

## References

[1] Crabtree PJ, Campana DV, Ryan K (1989). Early Animal Domestication and Its Cultural Context.

[2] Larson G, Albarella U, Dobney K, Rowley-Conwy P, Schibler J, Tresset A, Vigne J-D, Edwards CJ, Schlumbaum A, Dinu A (2007). Ancient DNA, pig domestication, and the spread of the Neolithic into Europe. Proc. Natl. Acad. Sci..

[3] Szűcs I, Vida V (2017). Global tendencies in pork meat-production, trade and consumption. Appl. Stud. Agribusiness Commerce.

[4] Kim J, Cho S, Caetano-Anolles K, Kim H, Ryu Y-C (2015). Genome-wide detection and characterization of positive selection in Korean Native Black Pig from Jeju Island. BMC Genet..

[5] Lee Y-S, Shin D, Won K-H, Kim DC, Lee SC, Song K-D (2020). Genome-wide scans for detecting the selection signature of the Jeju-island native pig in Korea. Asian Australas. J. Anim. Sci..

[6] Choi Y-S, Park B-Y, Lee J-M, Lee S-K (2005). Comparison of carcass and meat quality characteristics between Korean native black pigs and commercial crossbred pigs. Food Sci. Anim. Resour..

[7] Cho I-C, Park H-B, Ahn JS, Han S-H, Lee J-B, Lim H-T, Yoo C-K, Jung E-J, Kim D-H, Sun W-S (2019). A functional regulatory variant of MYH3 influences muscle fiber-type composition and intramuscular fat content in pigs. PLoS Genet..

[8] Cho S, Park B, Kim J, Kim M, Seong P, Kim Y, Kim D, Ahn C (2007). Carcass yields and meat quality by live weight of Korean native black pigs. J. Anim. Sci. Technol..

[9] Hur S, Jeong T, Kim G, Jeong J, Cho I, Lim H, Kim B, Joo S (2013). Comparison of live performance and meat quality parameter of cross bred (Korean native black pig and landrace) pigs with different coat colors. Asian Australas. J. Anim. Sci..

[10] Ballweg IC, Frölich K, Fandrey E, Meyer HH, Kliem H (2014). Comparison of the meat quality of Turopolje, German Landrace × Turopolje and German Landrace × Pietrain pigs. Agric. Conspec. Sci..

[11] Wu F, Vierck K, DeRouchey J, O'Quinn T, Tokach M, Goodband R, Dritz S, Woodworth J (2017). A review of heavy weight market pigs: Status of knowledge and future needs assessment. Transl. Anim. Sci..

[12] Incheol Jo, B. K. *et al*. A study on the ability improvement of 'Nanchuk Matdon', a high meat breeding herd based on Jeju native pigs (2017).

[13] Canovas S, Ivanova E, Romar R, García-Martínez S, Soriano-Ubeda C, García-Vázquez FA, Saadeh H, Andrews S, Kelsey G, Coy P (2017). DNA methylation and gene expression changes derived from assisted reproductive technologies can be decreased by reproductive fluids. Elife.

[14] Plassais J, Kim J, Davis BW, Karyadi DM, Hogan AN, Harris AC, Decker B, Parker HG, Ostrander EA (2019). Whole genome sequencing of canids reveals genomic regions under selection and variants influencing morphology. Nat. Commun..

[15] Srikanth K, Kim N-Y, Park W, Kim J-M, Kim K-D, Lee K-T, Son J-H, Chai H-H, Choi J-W, Jang G-W (2019). Comprehensive genome and transcriptome analyses reveal genetic relationship, selection signature, and transcriptome landscape of small-sized Korean native Jeju horse. Sci. Rep..

[16] Sabeti PC, Varilly P, Fry B, Lohmueller J, Hostetter E, Cotsapas C, Xie X, Byrne EH, McCarroll SA, Gaudet R (2007). Genome-wide detection and characterization of positive selection in human populations. Nature.

[17] Chen H, Patterson N, Reich D (2010). Population differentiation as a test for selective sweeps. Genome Res..

[18] Liao Y, Smyth GK, Shi W (2014). featureCounts: An efficient general purpose program for assigning sequence reads to genomic features. Bioinformatics.

[19] Yin L, Zhang H, Tang Z, Xu J, Yin D, Zhang Z, Yuan X, Zhu M, Zhao S, Li X (2021). rMVP: A memory-efficient, visualization-enhanced, and parallel-accelerated tool for genome-wide association study. Genomics Proteomics Bioinform..

[20] Szklarczyk D, Gable AL, Lyon D, Junge A, Wyder S, Huerta-Cepas J, Simonovic M, Doncheva NT, Morris JH, Bork P (2018). STRING v11: Protein–protein association networks with increased coverage, supporting functional discovery in genome-wide experimental datasets. Nucleic Acids Res..

[21] Bird A (2002). DNA methylation patterns and epigenetic memory. Genes Dev..

[22] Hayes BJ, Bowman PJ, Chamberlain AC, Verbyla K, Goddard ME (2009). Accuracy of genomic breeding values in multi-breed dairy cattle populations. Genet. Sel. Evol..

[23] Zhuang Z, Li S, Ding R, Yang M, Zheng E, Yang H, Gu T, Xu Z, Cai G, Wu Z (2019). Meta-analysis of genome-wide association studies for loin muscle area and loin muscle depth in two Duroc pig populations. PLoS ONE.

[24] Cordero AIH, Gonzales NM, Parker CC, Sokolof G, Vandenbergh DJ, Cheng R, Abney M, Sko A, Douglas A, Palmer AA (2019). Genome-wide associations reveal human-mouse genetic convergence and modifiers of myogenesis, CPNE1 and STC2. Am. J. Hum. Genet..

[25] Willems SM, Wright DJ, Day FR, Trajanoska K, Joshi PK, Morris JA, Matteini AM, Garton FC, Grarup N, Oskolkov N (2017). Large-scale GWAS identifies multiple loci for hand grip strength providing biological insights into muscular fitness. Nat. Commun..

[26] Ponsuksili S, Murani E, Trakooljul N, Schwerin M, Wimmers K (2014). Discovery of candidate genes for muscle traits based on GWAS supported by eQTL-analysis. Int. J. Biol. Sci..

[27] Park W, Kim J, Kim HJ, Choi J, Park J-W, Cho H-W, Kim B-W, Park MH, Shin T-S, Cho S-K (2014). Investigation of de novo unique differentially expressed genes related to evolution in exercise response during domestication in Thoroughbred race horses. PLoS ONE.

[28] Zhao P, Yu Y, Feng W, Du H, Yu J, Kang H, Zheng X, Wang Z, Liu GE, Ernst CW (2018). Evidence of evolutionary history and selective sweeps in the genome of Meishan pig reveals its genetic and phenotypic characterization. GigaScience.

[29] Wang K, Wu P, Yang Q, Chen D, Zhou J, Jiang A, Ma J, Tang Q, Xiao W, Jiang Y (2018). Detection of selection signatures in Chinese Landrace and Yorkshire pigs based on genotyping-by-sequencing data. Front. Genet..

[30] Listrat A, Lebret B, Louveau I, Astruc T, Bonnet M, Lefaucheur L, Picard B, Bugeon J (2016). How muscle structure and composition influence meat and flesh quality. Sci. World J..

[31] Kim H, Song KD, Kim HJ, Park W, Kim J, Lee T, Shin D-H, Kwak W, Kwon Y-J, Sung S (2015). Exploring the genetic signature of body size in Yucatan miniature pig. PLoS ONE.

[32] Bakker EG, Traw MB, Toomajian C, Kreitman M, Bergelson J (2008). Low levels of polymorphism in genes that control the activation of defense response in *Arabidopsis**thaliana*. Genetics.

[33] VanBuren R, Wai CM, Zhang J, Han J, Arro J, Lin Z, Liao Z, Yu Q, Wang M-L, Zee F (2016). Extremely low nucleotide diversity in the X-linked region of papaya caused by a strong selective sweep. Genome Biol..

[34] Meech R, Mubarokah N, Shivasami A, Rogers A, Nair PC, Hu DG, McKinnon RA, Mackenzie PI (2015). A novel function for UDP glycosyltransferase 8: Galactosidation of bile acids. Mol. Pharmacol..

[35] Chiang JY, Ferrell JM (2018). Bile acid metabolism in liver pathobiology. Gene Expr. J. Liver Res..

[36] Huang DW, Sherman BT, Lempicki RA (2009). Bioinformatics enrichment tools: Paths toward the comprehensive functional analysis of large gene lists. Nucleic Acids Res..

[37] Ganguly R, Mohyeldin A, Thiel J, Kornblum HI, Beullens M, Nakano I (2015). MELK—A conserved kinase: Functions, signaling, cancer, and controversy. Clin. Transl. Med..

[38] Nakano I, Paucar AA, Bajpai R, Dougherty JD, Zewail A, Kelly TK, Kim KJ, Ou J, Groszer M, Imura T (2005). Maternal embryonic leucine zipper kinase (MELK) regulates multipotent neural progenitor proliferation. J. Cell Biol..

[39] Ahbara A, Bahbahani H, Almathen F, Al Abri M, Agoub MO, Abeba A, Kebede A, Musa HH, Mastrangelo S, Pilla F (2019). Genome-wide variation, candidate regions and genes associated with fat deposition and tail morphology in Ethiopian indigenous sheep. Front. Genet..

[40] Ramayo-Caldas Y, Ballester M, Fortes MR, Esteve-Codina A, Castelló A, Noguera JL, Fernández AI, Pérez-Enciso M, Reverter A, Folch JM (2014). From SNP co-association to RNA co-expression: Novel insights into gene networks for intramuscular fatty acid composition in porcine. BMC Genomics.

[41] Khan I, Steeg PS (2018). The relationship of NM23 (NME) metastasis suppressor histidine phosphorylation to its nucleoside diphosphate kinase, histidine protein kinase and motility suppression activities. Oncotarget.

[42] Shibata E, Abbas T, Huang X, Wohlschlegel JA, Dutta A (2011). Selective ubiquitylation of p21 and Cdt1 by UBCH8 and UBE2G ubiquitin-conjugating enzymes via the CRL4Cdt2 ubiquitin ligase complex. Mol. Cell. Biol..

[43] Buchwald M, Pietschmann K, Müller J, Böhmer F, Heinzel T, Krämer O (2010). Ubiquitin conjugase UBCH8 targets active FMS-like tyrosine kinase 3 for proteasomal degradation. Leukemia.

[44] Marcelin G, Liu S-M, Schwartz GJ, Chua SC (2013). Identification of a loss-of-function mutation in Ube2l6 associated with obesity resistance. Diabetes.

[45] Padanad MS, Konstantinidou G, Venkateswaran N, Melegari M, Rindhe S, Mitsche M, Yang C, Batten K, Huffman KE, Liu J (2016). Fatty acid oxidation mediated by Acyl-CoA synthetase long chain 3 is required for mutant KRAS lung tumorigenesis. Cell Rep..

[46] Dragoš A, Kovács ÁT (2017). The peculiar functions of the bacterial extracellular matrix. Trends Microbiol..

[47] Frantz C, Stewart KM, Weaver VM (2010). The extracellular matrix at a glance. J. Cell Sci..

[48] Green YS, Vetter ML (2011). EBF factors drive expression of multiple classes of target genes governing neuronal development. Neural Dev..

[49] Jin S, Kim J, Willert T, Klein-Rodewald T, Garcia-Dominguez M, Mosqueira M, Fink R, Esposito I, Hofbauer LC, Charnay P (2014). Ebf factors and MyoD cooperate to regulate muscle relaxation via Atp2a1. Nat. Commun..

[50] Wu T, Zhang X, Tian M, Tao Q, Zhang L, Ding Y, Zhang X, Yin Z (2018). Transcriptome analysis reveals candidate genes involved in splay leg syndrome in piglets. J. Appl. Genet..

[51] Percie du Sert N, Hurst V, Ahluwalia A, Alam S, Avey MT, Baker M, Browne WJ, Clark A, Cuthill IC, Dirnagl U (2020). The ARRIVE guidelines 2.0: Updated guidelines for reporting animal research. J. Cereb. Blood Flow Metab..

[52] Ghosh M, Sodhi SS, Sharma N, Mongre RK, Kim N, Singh AK, Lee SJ, Kim DC, Kim SW, Lee HK (2016). An integrated in silico approach for functional and structural impact of non-synonymous SNPs in the MYH1 gene in Jeju Native Pigs. BMC Genet..

[53] Li H, Durbin R (2009). Fast and accurate short read alignment with Burrows-Wheeler transform. Bioinformatics.

[54] Li H, Handsaker B, Wysoker A, Fennell T, Ruan J, Homer N, Marth G, Abecasis G, Durbin R (2009). The sequence alignment/map format and SAMtools. Bioinformatics.

[55] De Summa S, Malerba G, Pinto R, Mori A, Mijatovic V, Tommasi S (2017). GATK hard filtering: Tunable parameters to improve variant calling for next generation sequencing targeted gene panel data. BMC Bioinform.

[56] Andrews, S. F., Krueger, F., Seconds-Pichon, A., Biggins, F. & Wingett. S. F. A quality control tool for high throughput sequence data. *Babraham**Bioinformatics* (2014).

[57] Poplin R, Ruano-Rubio V, DePristo MA, Fennell TJ, Carneiro MO, Van der Auwera GA, Kling DE, Gauthier LD, Levy-Moonshine A, Roazen D (2017). Scaling accurate genetic variant discovery to tens of thousands of samples. bioRxiv.

[58] Ren S, Bertels K, Al-Ars Z (2018). Efficient acceleration of the pair-hmms forward algorithm for gatk haplotypecaller on graphics processing units. Evol. Bioinform..

[59] Matika O, Robledo D, Pong-Wong R, Bishop SC, Riggio V, Finlayson H, Lowe NR, Hoste AE, Walling GA, del Pozo J (2019). Balancing selection at a premature stop mutation in the myostatin gene underlies a recessive leg weakness syndrome in pigs. PLoS Genet..

[60] Browning SR, Browning BL (2007). Rapid and accurate haplotype phasing and missing-data inference for whole-genome association studies by use of localized haplotype clustering. Am. J. Hum. Genet..

[61] Zheng X (2013). A Tutorial for the R Package SNPRelate.

[62] Zheng X, Levine D, Shen J, Gogarten SM, Laurie C, Weir BS (2012). A high-performance computing toolset for relatedness and principal component analysis of SNP data. Bioinformatics.

[63] Yu G (2020). Using ggtree to visualize data on tree-like structures. Curr. Protoc. Bioinform..

[64] Patterson N, Moorjani P, Luo Y, Mallick S, Rohland N, Zhan Y, Genschoreck T, Webster T, Reich D (2012). Ancient admixture in human history. Genetics.

[65] Liu X, Ong RT-H, Pillai EN, Elzein AM, Small KS, Clark TG, Kwiatkowski DP, Teo Y-Y (2013). Detecting and characterizing genomic signatures of positive selection in global populations. Am. J. Hum. Genet..

[66] Fu W, Lee WR, Abasht B (2016). Detection of genomic signatures of recent selection in commercial broiler chickens. BMC Genet..

[67] Hrdlickova R, Toloue M, Tian B (2017). RNA-Seq methods for transcriptome analysis. Wiley Interdiscip. Rev. RNA.

[68] Kukurba KR, Montgomery SB (2015). RNA sequencing and analysis. Cold Spring Harbor Protoc..

[69] Lowe R, Shirley N, Bleackley M, Dolan S, Shafee T (2017). Transcriptomics technologies. PLoS Comput. Biol..

[70] Costa-Silva J, Domingues D, Lopes FM (2017). RNA-Seq differential expression analysis: An extended review and a software tool. PLoS ONE.

[71] Bolger AM, Lohse M (2014). Usadel BJB: Trimmomatic: A flexible trimmer for Illumina sequence data. Bioinformatics.

[72] Fujita PA, Rhead B, Zweig AS, Hinrichs AS, Karolchik D, Cline MS, Goldman M, Barber GP, Clawson H, Coelho A (2010). The UCSC genome browser database: Update 2011. Nucleic Acids Res..

[73] Kim D, Langmead B, Salzberg SL (2015). HISAT: A fast spliced aligner with low memory requirements. Nat. Methods.

[74] Love MI, Huber W, Anders S (2014). Moderated estimation of fold change and dispersion for RNA-seq data with DESeq2. Genome Biol..

[75] Ashburner M, Ball CA, Blake JA, Botstein D, Butler H, Cherry JM, Davis AP, Dolinski K, Dwight SS, Eppig JT (2000). Gene ontology: Tool for the unification of biology. Nat. Genet..

[76] Supek F, Bošnjak M, Škunca N, Šmuc T (2011). REVIGO summarizes and visualizes long lists of gene ontology terms. PLoS ONE.

[77] Durinck S, Moreau Y, Kasprzyk A, Davis S, De Moor B, Brazma A, Huber W (2005). BioMart and Bioconductor: A powerful link between biological databases and microarray data analysis. Bioinformatics.

[78] Smedley D, Haider S, Ballester B, Holland R, London D, Thorisson G, Kasprzyk A (2009). BioMart—biological queries made easy. BMC Genomics.

[79] Sherman BT, Lempicki RA (2009). Systematic and integrative analysis of large gene lists using DAVID bioinformatics resources. Nat. Protoc..

[80] Kanehisa M, Sato Y, Kawashima M, Furumichi M, Tanabe M (2016). KEGG as a reference resource for gene and protein annotation. Nucleic Acids Res..

[81] Yu G, Wang L-G, Han Y, He Q-Y (2012). clusterProfiler: An R package for comparing biological themes among gene clusters. OMICS.

[82] Wurmus R, Uyar B, Osberg B, Franke V, Gosdschan A, Wreczycka K, Ronen J, Akalin A (2018). PiGx: Reproducible genomics analysis pipelines with GNU Guix. Gigascience.

[83] Krueger F (2015). Trim galore. A wrapper tool around Cutadapt and FastQC to consistently apply quality and adapter trimming to FastQ files.

[84] Krueger F, Andrews SR (2011). Bismark: A flexible aligner and methylation caller for Bisulfite-Seq applications. Bioinformatics.

