# Supplementary material for: Integration of multi-omics approaches for functional characterization of muscle related selective sweep genes in Nanchukmacdon
Source: Sci Rep. 2021 Mar 30;11:7219. doi: 10.1038/s41598-021-86683-4 (PMC8009959; doi:10.1038/s41598-021-86683-4)
Supplement: Supplementary file 4 — Supplementary Information 4. [file 41598_2021_86683_MOESM4_ESM.docx]

| **ENS_ID** | **Chr** | **XP-CLR** | **XP-EHH** | **log2FC** | **FDR** | **Gene Symbol** | **Compare** |
| --- | --- | --- | --- | --- | --- | --- | --- |
| ENSSSCG00000021562 | 18 | 79.58527 | -2.085748 | 1.21 | 0.005128 | *PLXNA4* | Nanchukmacdon  VS  Jeju Native Pig |
| ENSSSCG00000006199 | 4 | 84.57209 | -2.527059 | 1.3 | 0.000426 | *PREX2* |  |
| ENSSSCG00000014255 | 2 | 62.42468 | -2.114777 | 1.72 | 5.59E-07 | *SLC12A2* |  |
| ENSSSCG00000009125 | 8 | 46.31273 | -2.22322 | 1.07 | 7.09E-11 | *ANK2* | Nanchukmacdon  VS  Duroc |
| ENSSSCG00000006878 | 4 | 43.71326 | -2.23917 | 1.09 | 2.69E-10 | *DPYD* |  |
| ENSSSCG00000006039 | 4 | 61.13748 | -3.18808 | 1.09 | 1.79E-08 | *LRP12* |  |
| ENSSSCG00000005444 | 1 | 67.20646 | -2.40928 | 1.64 | 6.14E-58 | *TMEM245* |  |
| ENSSSCG00000027074 | 2 | 70.466 | -2.12986 | 1.82 | 3.34E-27 | *FER* |  |
| ENSSSCG00000017095 | 16 | 43.00722 | -2.00127 | 2.94 | 3.86E-19 | *SEMA5A* |  |
| ENSSSCG00000006039 | 4 | 55.49041 | -2.53032 | 1.2 | 2.47E-13 | *LRP12* | Nanchukmacdon  VS  Landrace |
| ENSSSCG00000038801 | 8 | 60.31172 | -2.15324 | 1.77 | 5.5E-06 | *NPNT* |  |
| ENSSSCG00000030378 | 3 | 107.8206 | -2.72439 | 2.52 | 7.36E-29 | *LIMK1* |  |
| ENSSSCG00000016160 | 15 | 71.99927 | -2.40967 | 3.02 | 2.29E-11 | *ERBB4* |  |

Additional table 1: Commonly identified selective signature genes with top 1% of XP-CLR and -2 cutoff for XP-EHH score with log2fold change of < 1 and FDR of 0.05 against JNP, Duroc and Landrace.
