# Supplementary material for: Integration of multi-omics approaches for functional characterization of muscle related selective sweep genes in Nanchukmacdon
Source: Sci Rep. 2021 Mar 30;11:7219. doi: 10.1038/s41598-021-86683-4 (PMC8009959; doi:10.1038/s41598-021-86683-4)
Supplement: Supplementary file 5 — Supplementary Information 5. [file 41598_2021_86683_MOESM5_ESM.docx]

| **Category** | **Term** | **Count** | **%** | **PValue** | **Compare** |
| --- | --- | --- | --- | --- | --- |
| BP | GO:0030198~extracellular matrix organization | 15 | 1.017639077 | 1.62E-05 | Nanchukmacdon  VS  Jeju Native Pig |
|  | GO:0007018~microtubule-based movement | 13 | 0.881953867 | 2.65E-04 |  |
|  | GO:0001578~microtubule bundle formation | 7 | 0.474898236 | 0.001387 |  |
| CC | GO:0005578~proteinaceous extracellular matrix | 30 | 2.035278155 | 4.86E-06 |  |
|  | GO:0005871~kinesin complex | 13 | 0.881953867 | 3.73E-05 |  |
|  | GO:0031012~extracellular matrix | 20 | 1.356852103 | 1.28E-04 |  |
| MF | GO:0005509~calcium ion binding | 63 | 4.274084125 | 7.64E-05 |  |
|  | GO:0008201~heparin binding | 17 | 1.153324288 | 7.28E-04 |  |
|  | GO:0003777~microtubule motor activity | 11 | 0.746268657 | 0.001807 |  |
| BP | GO:0030198~extracellular matrix organization | 17 | 0.763016158 | 1.47E-04 | Nanchukmacdon  VS  Duroc |
|  | GO:0045766~positive regulation of angiogenesis | 21 | 0.942549372 | 2.03E-04 |  |
|  | GO:0000381~regulation of alternative mRNA splicing, via spliceosome | 12 | 0.538599641 | 3.33E-04 |  |
| CC | GO:0005737~cytoplasm | 364 | 16.33752244 | 5.94E-10 |  |
|  | GO:0070062~extracellular exosome | 274 | 12.29802513 | 3.71E-08 |  |
|  | GO:0005654~nucleoplasm | 172 | 7.719928187 | 1.03E-07 |  |
| MF | GO:0000166~nucleotide binding | 54 | 2.423698384 | 7.63E-06 |  |
|  | GO:0050840~extracellular matrix binding | 9 | 0.403949731 | 8.62E-04 |  |
|  | GO:0005509~calcium ion binding | 84 | 3.770197487 | 9.39E-04 |  |
| BP | GO:0030199~collagen fibril organization | 13 | 0.617283951 | 4.22E-06 | Nanchukmacdon  VS  Landrace |
|  | GO:0007018~microtubule-based movement | 18 | 0.854700855 | 1.15E-05 |  |
|  | GO:0001525~angiogenesis | 26 | 1.234567901 | 1.24E-04 |  |
| CC | GO:0070062~extracellular exosome | 272 | 12.91547958 | 8.86E-10 |  |
|  | GO:0005578~proteinaceous extracellular matrix | 44 | 2.089268756 | 7.08E-09 |  |
|  | GO:0031012~extracellular matrix | 29 | 1.377018044 | 1.61E-06 |  |
| MF | GO:0005509~calcium ion binding | 105 | 4.985754986 | 1.46E-10 |  |
|  | GO:0005201~extracellular matrix structural constituent | 14 | 0.664767331 | 5.99E-06 |  |
|  | GO:0005524~ATP binding | 162 | 7.692307692 | 1.30E-05 |  |
