# Supplementary material for: Integration of multi-omics approaches for functional characterization of muscle related selective sweep genes in Nanchukmacdon
Source: Sci Rep. 2021 Mar 30;11:7219. doi: 10.1038/s41598-021-86683-4 (PMC8009959; doi:10.1038/s41598-021-86683-4)
Supplement: Supplementary file 6 — Supplementary Information 6. [file 41598_2021_86683_MOESM6_ESM.docx]

| **No.** | **Name** | **Sequence (5'->3')** | **Tm** | **GC%** |
| --- | --- | --- | --- | --- |
| 1 | Sus_GAPDH_F | GGCGTGAACCATGAGAAGTAT | 55.9 | 48 |
|  | Sus_GAPDH_R | CCCTCCACGATGCCGAAGT | 59.2 | 63 |
| 2 | Sus_beta-actin_F | CACGCCATCCTGCGTCTGGA | 63.4 | 65 |
|  | Sus_beta-actin_R | AGCACCGTGTTGGCGTAGAG | 61.4 | 60 |
| 3 | UGT8_F | ACATCGCGCCATCTCATCAT | 57.3 | 50 |
|  | UGT8_R | GCAGGATACCAAAGGCCAGT | 59.3 | 55 |
| 4 | UBE2L6_F | AAACCGCCCTACAACCTCAG | 59.3 | 55 |
|  | UBE2L6_R | AGGGCTTCCAGTTCTCGTTG | 59.3 | 55 |
| 5 | MELK_F | GCCCTCAGAGTCCACATCCTT | 61.8 | 57 |
|  | MELK_R | CGGAACACAACTCGGGTCTC | 61.4 | 60 |
| 6 | ZGRF1_F | GTAGATGCTTTTCAGGGCGC | 59.3 | 55 |
|  | ZGRF1_R | TCCTTCCTCTGGTCAATGCA | 57.3 | 55 |
| 7 | NCALD_F | TGGTTTCTGTCTTGCTTGCAG | 57.9 | 48 |
|  | NCALD_R | GTGCGCACATGCAAGAGATT | 57.3 | 50 |
| 8 | FHL2_F | AATGAAGGGTTGGTGTCAGG | 57.3 | 50 |
|  | FHL2_R | CGGGTATTGGCTTGATGTG | 56.7 | 53 |
| 9 | SERP2_F | AGAAAAAAAGGCTCCCTCCA | 55.2 | 45 |
|  | SERP2_R | CAATCCTGCCAAGAGCAAAG | 57.3 | 50 |
