# Supplementary material for: Integration of multi-omics approaches for functional characterization of muscle related selective sweep genes in Nanchukmacdon
Source: Sci Rep. 2021 Mar 30;11:7219. doi: 10.1038/s41598-021-86683-4 (PMC8009959; doi:10.1038/s41598-021-86683-4)
Supplement: Supplementary file 7 — Supplementary Information 7. [file 41598_2021_86683_MOESM7_ESM.docx]

| **Sample Name** | **Sex** | **Number of Nipples** | | **Date of Birth**  **Age** | **Weight (Kg)** | | | **HpyCH4** | **SCD** | **Black Hair** | **Rib** | **Selection & Date** |
| --- | --- | --- | --- | --- | --- | --- | --- | --- | --- | --- | --- | --- |
|  | **♀/♂** | **Right** | **Left** |  | **Birth** | **Weaning period** | **70days** |  |  |  |  |  |
| F10-63 | ♂ | 8 | 8 | 2018-02-26 | 1.4 | 3.4 | 28 | 12 | AG | 12 | AG | Non castration, 2020/04/19 |
| F10-65 | ♂ | 7 | 7 | 2018-02-26 | 1.4 | 3.8 | 28 | 12 | AG | 12 | AG | Non castration, 2020/04/19 |
| F10-67 | ♂ | 8 | 8 | 2018-02-26 | 1.0 | 2.4 | 28 | 12 | AG | 12 | AG | Non castration, 2020/04/19 |
| F10-69 | ♂ | 7 | 7 | 2018-02-26 | 1.6 | 3.8 | 28 | 12 | AG | 12 | GG | Non castration, 2020/04/19 |
| F10-71 | ♂ | 8 | 7 | 2018-02-26 | 1.2 | 2.8 | 28 | 12 | AG | 12 | GG | Non castration, 2020/04/19 |
| F10-72 | ♂ | 7 | 7 | 2018-02-26 | 1.4 | 2.6 | 28 | 12 | AG | 12 | AG | Non castration, 2020/04/19 |
| F10-268 | ♂ | 6 | 6 | 2018-04-24 | 1.8 | 5.4 | 29 | 12 | A/G | 12 | A/A | test slaughter, 2020/06/06 |
| F10-272 | ♂ | 6 | 6 | 2017-12-20 | 1.6 | 5.8 | 29 | 12 | A/G | 12 | A/G | test slaughter, 2020/06/06 |
| F10-278 | ♂ | 6 | 6 | 2018-04-25 | 1.6 | 5.6 | 29 | 11 | A/A | 22 | A/G | test slaughter, 2020/06/06 |
| F10-284 | ♂ | 6 | 7 | 2017-12-20 | 1.6 | 6.2 | 29 | 11 | A/A | 22 | A/G | test slaughter, 2020/06/06 |
| F10-30 | ♂ | 6 | 6 | 2017-12-20 | 1.8 | 6.2 | 28 | 22 | AG | 22 | AA | test slaughter, 2020/06/06 |
