# Supplementary figures and images for: Integration of multi-omics approaches for functional characterization of muscle related selective sweep genes in Nanchukmacdon
Source: Sci Rep. 2021 Mar 30;11:7219. doi: 10.1038/s41598-021-86683-4 (PMC8009959; doi:10.1038/s41598-021-86683-4)

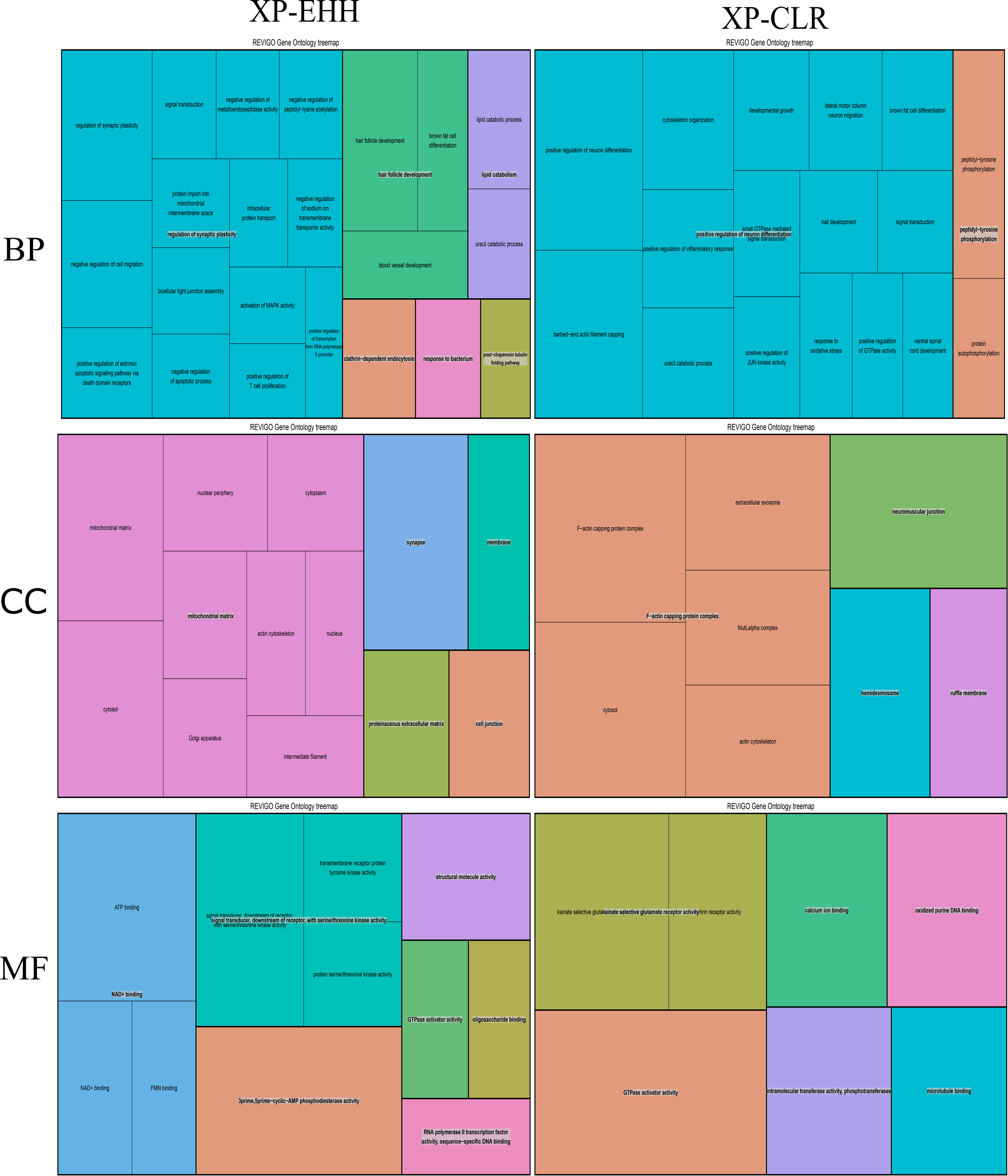

Supplement: Supplementary file 8 — Supplementary Information 8. [file 41598_2021_86683_MOESM8_ESM.tif]

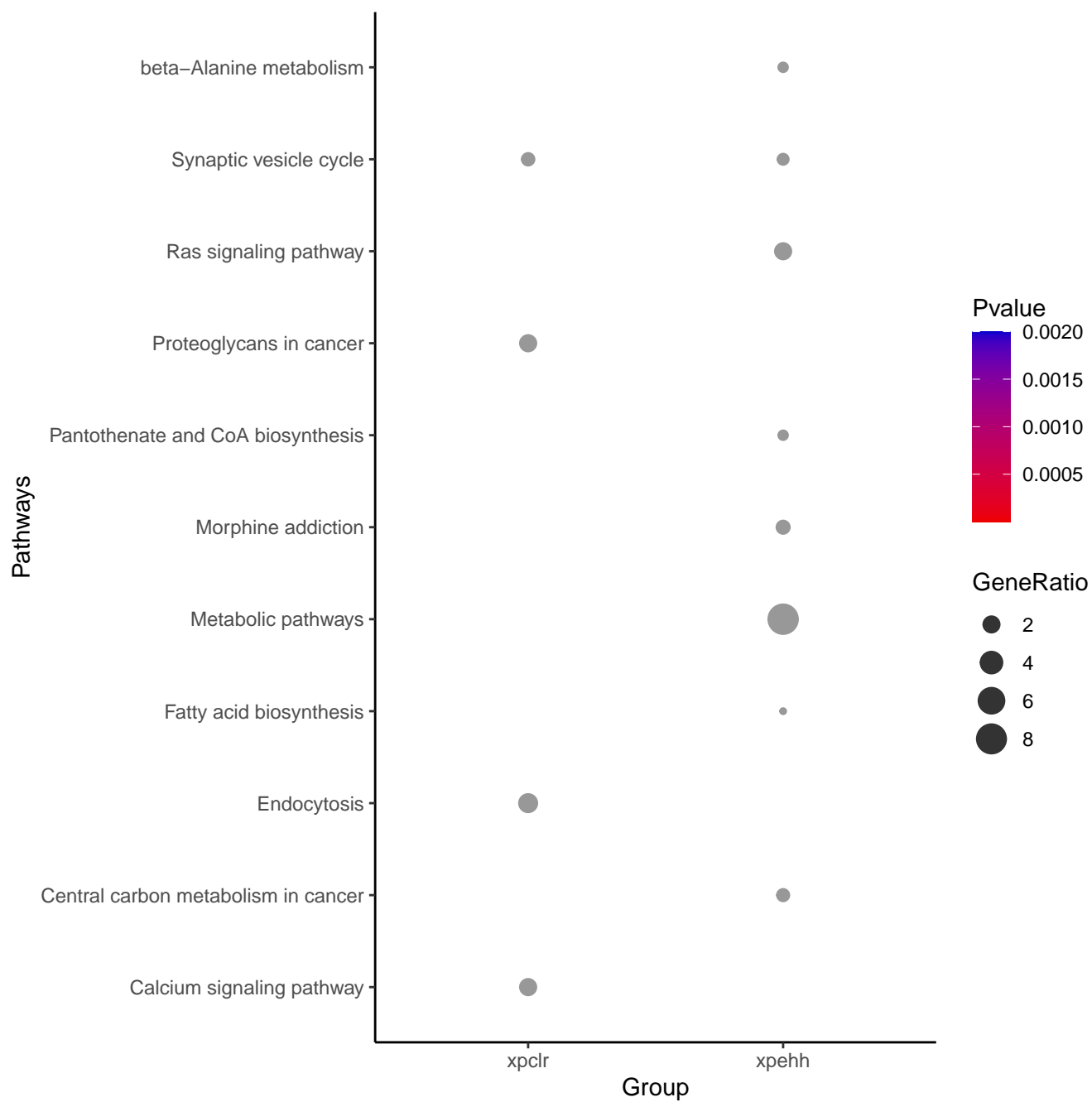

Supplement: Supplementary file 10 — Supplementary Information 10. [file 41598_2021_86683_MOESM10_ESM.pdf]

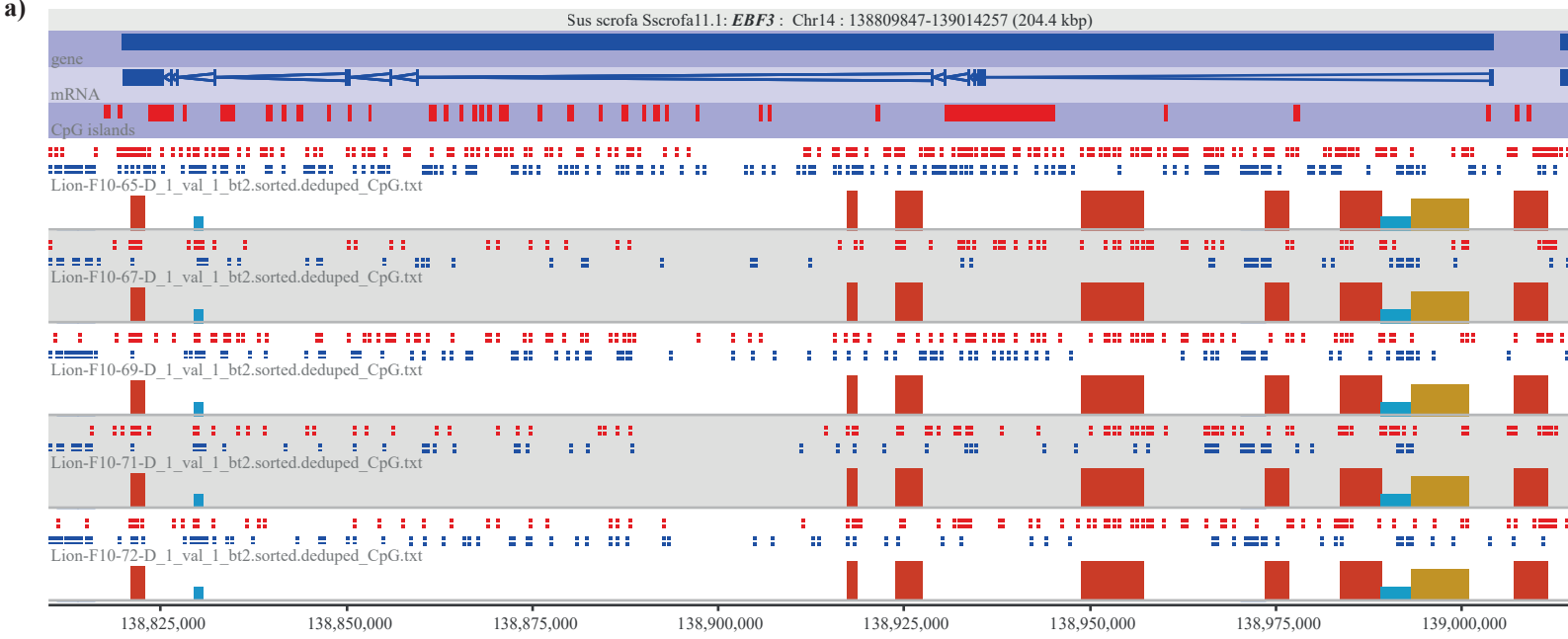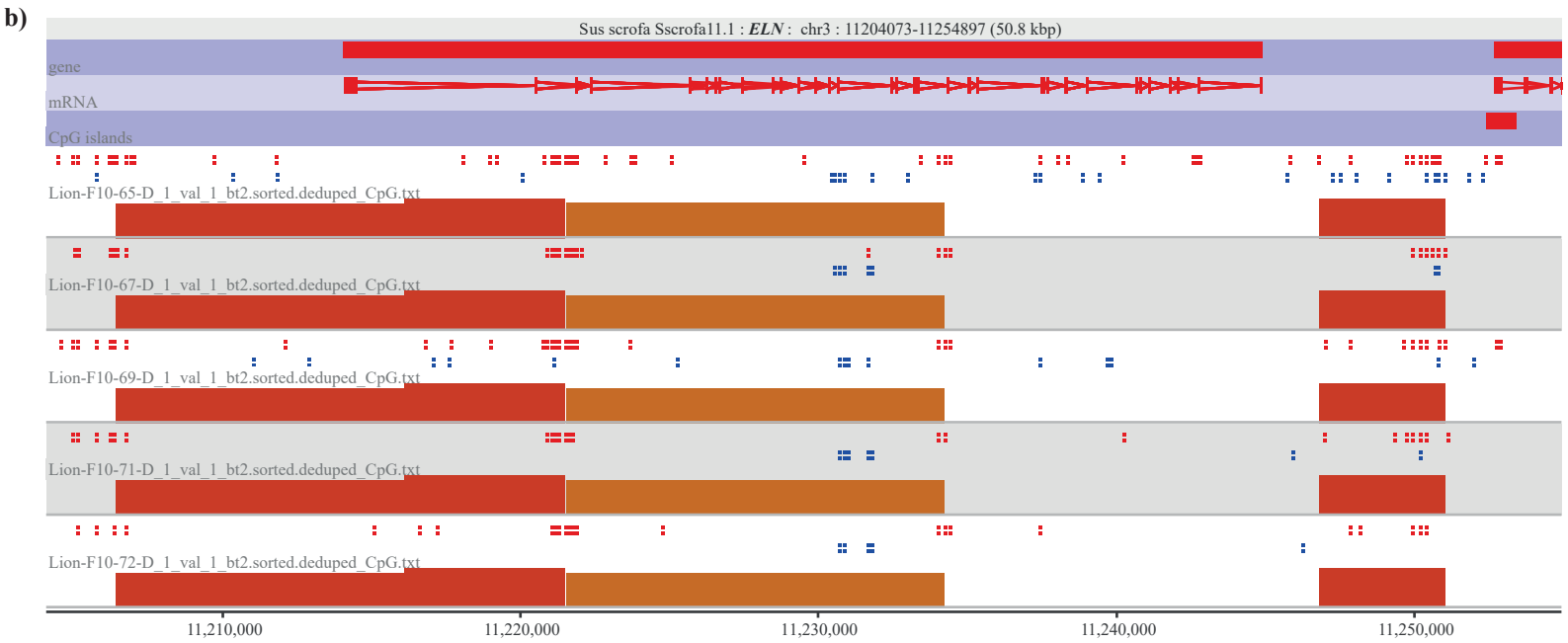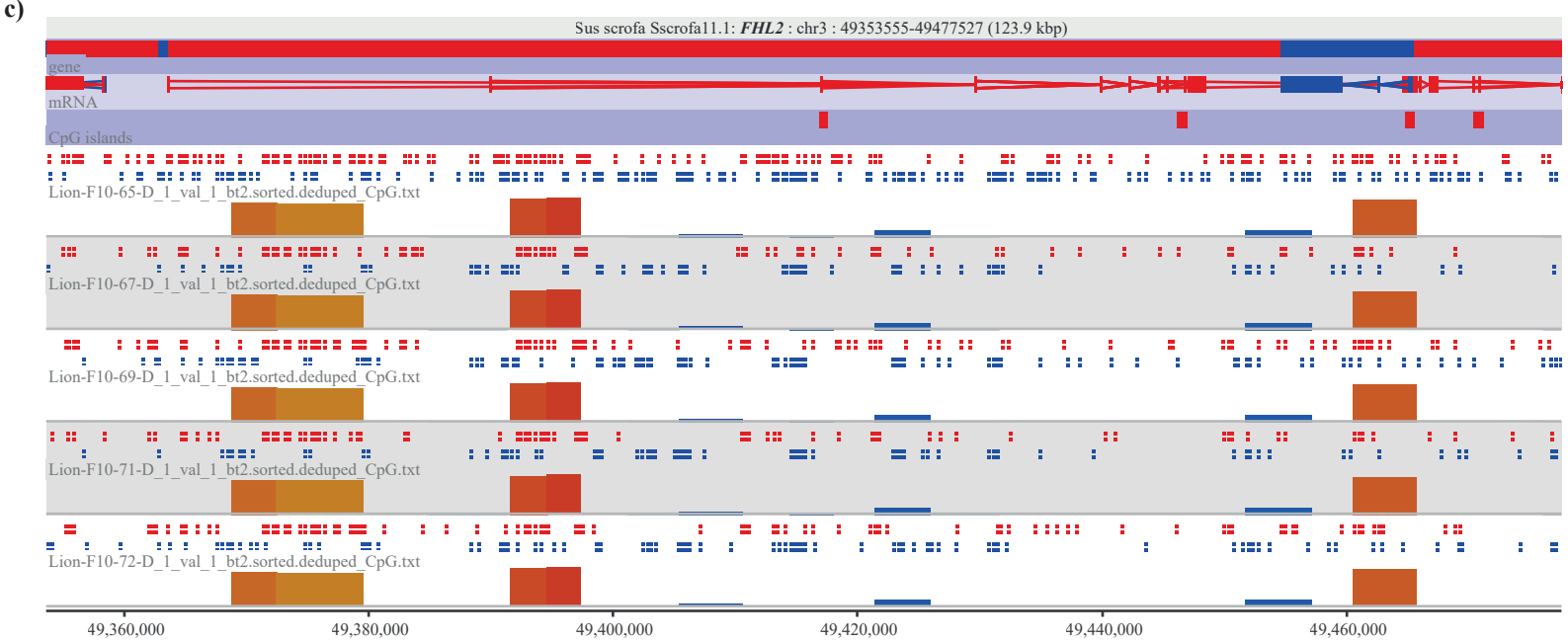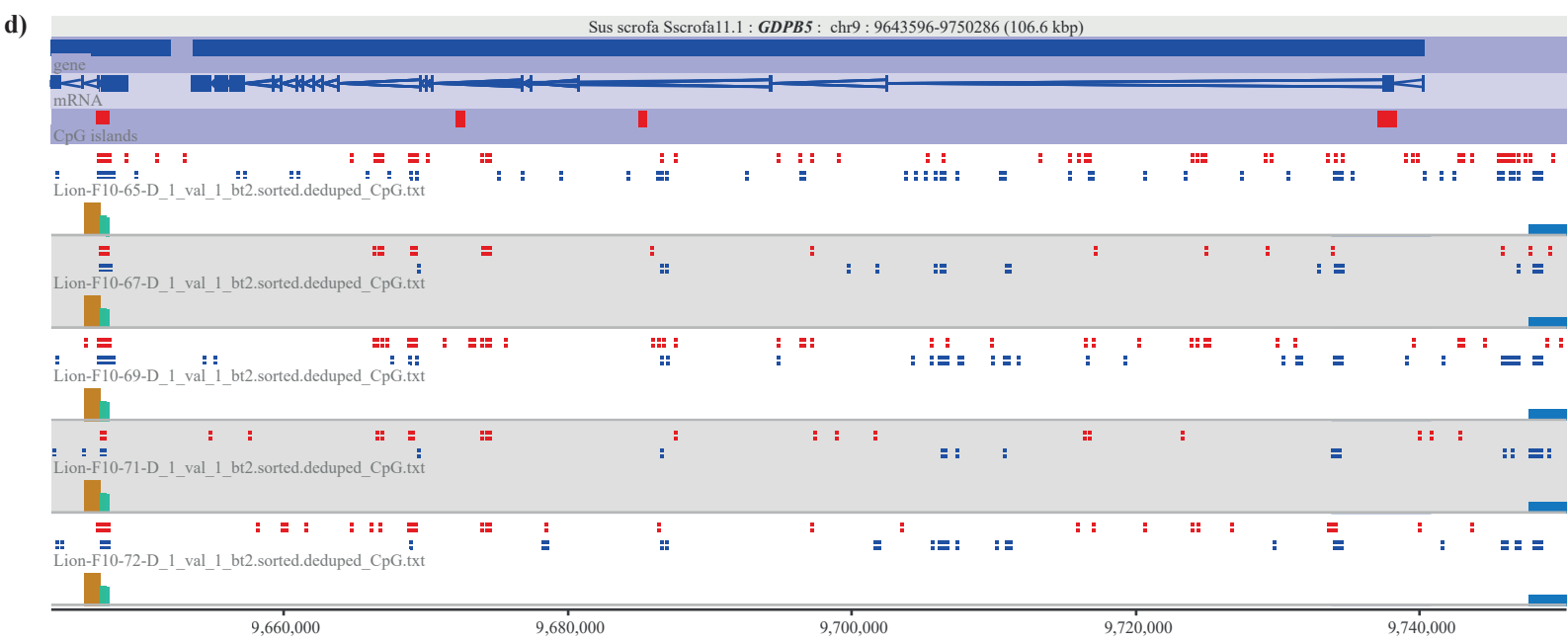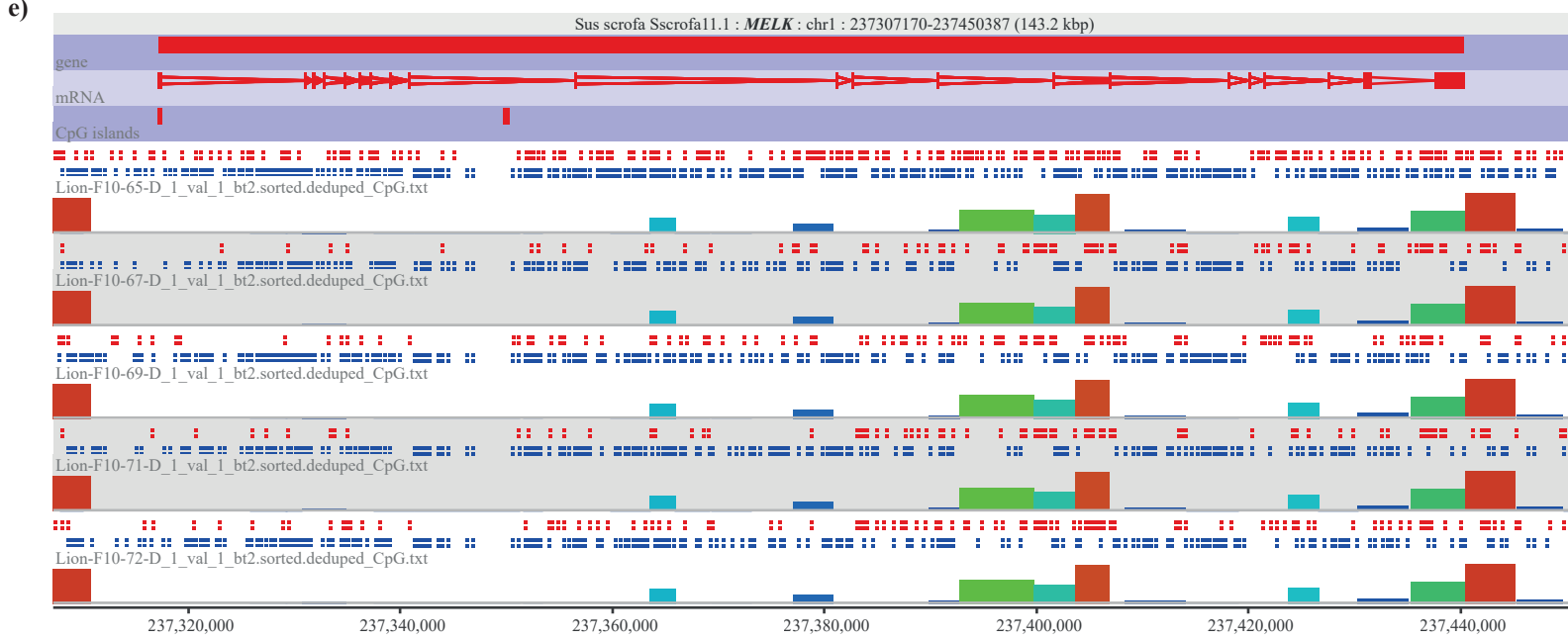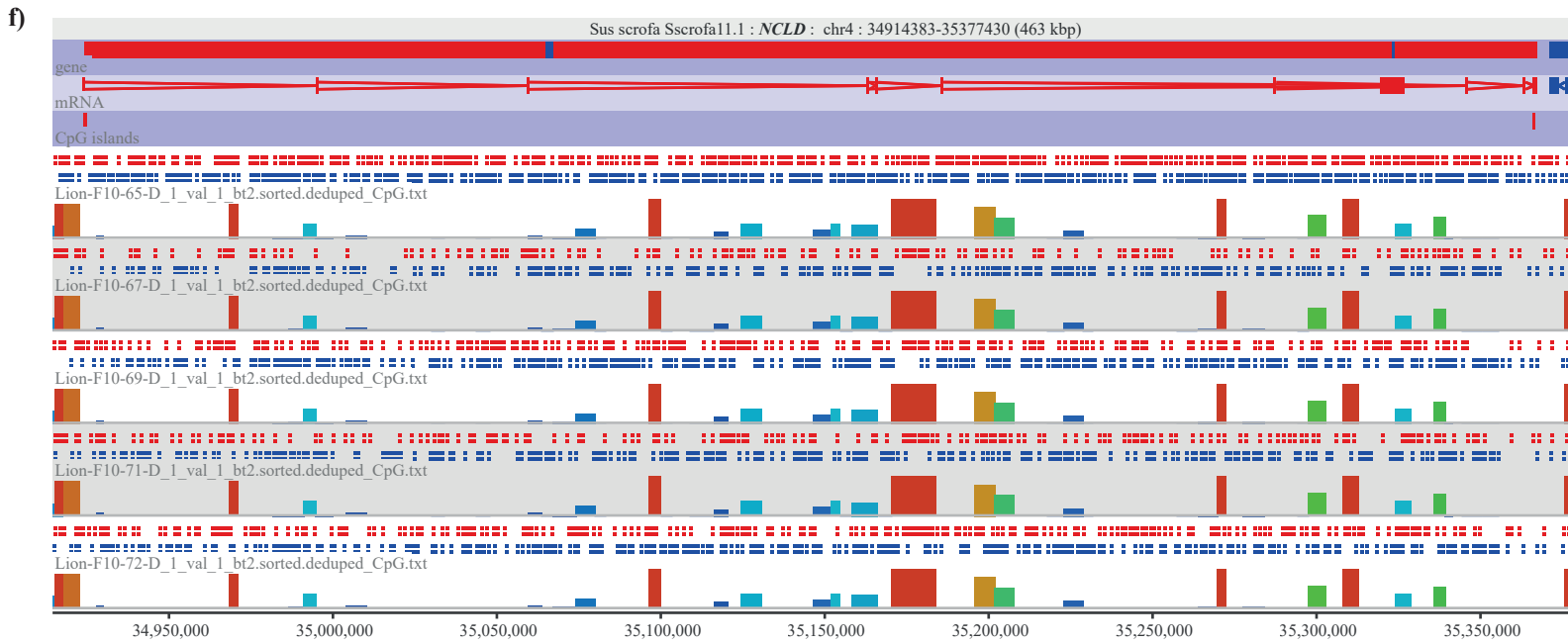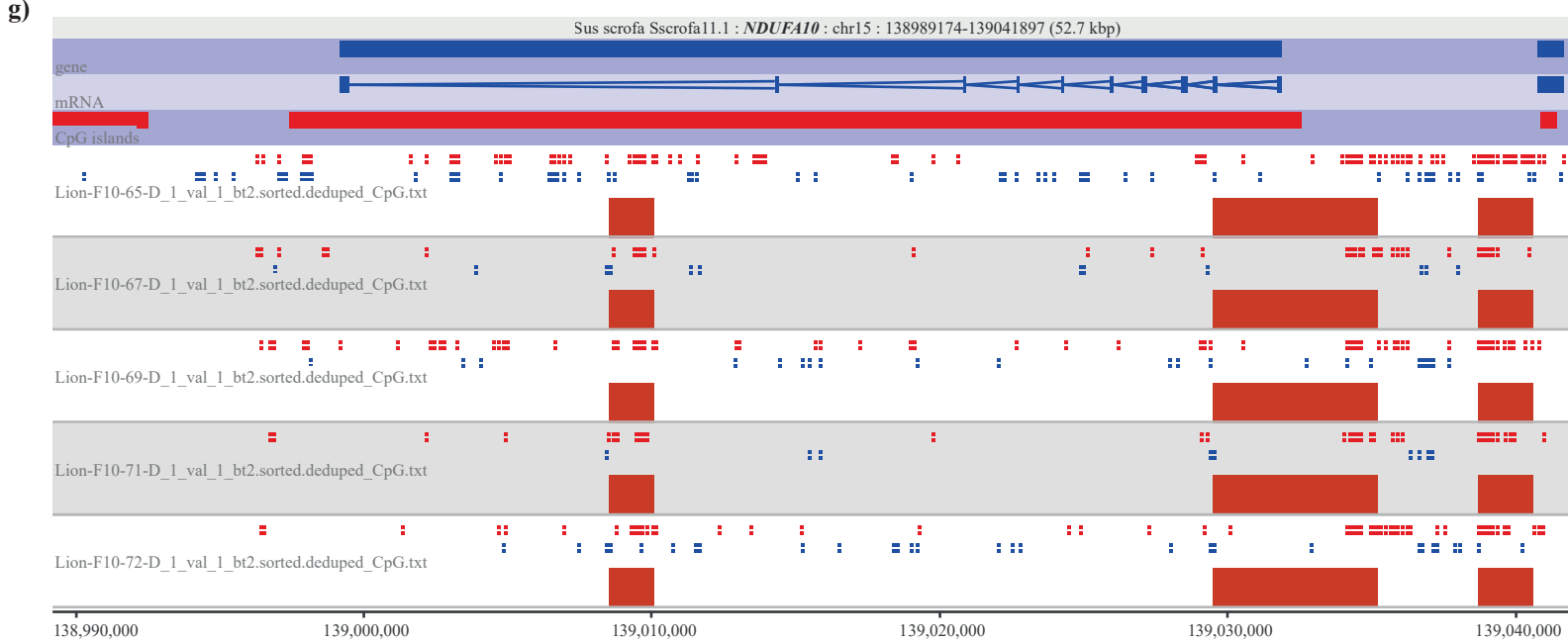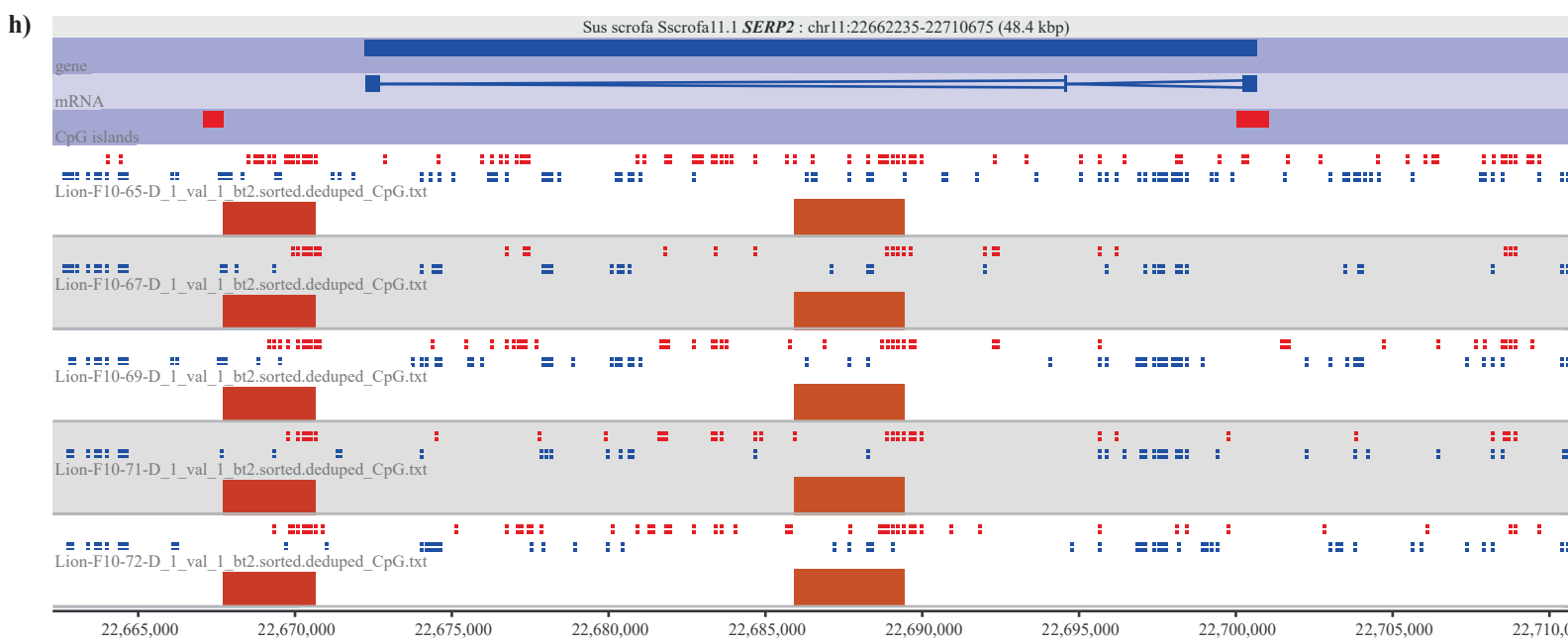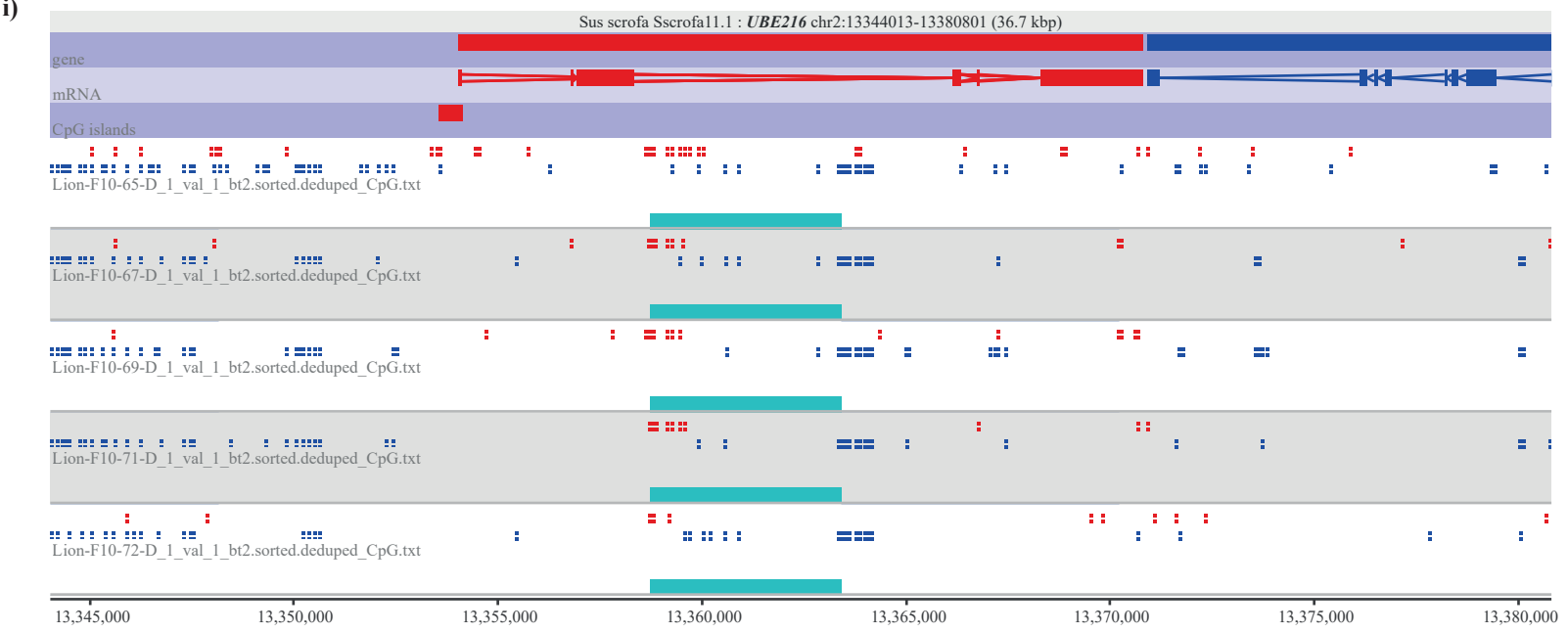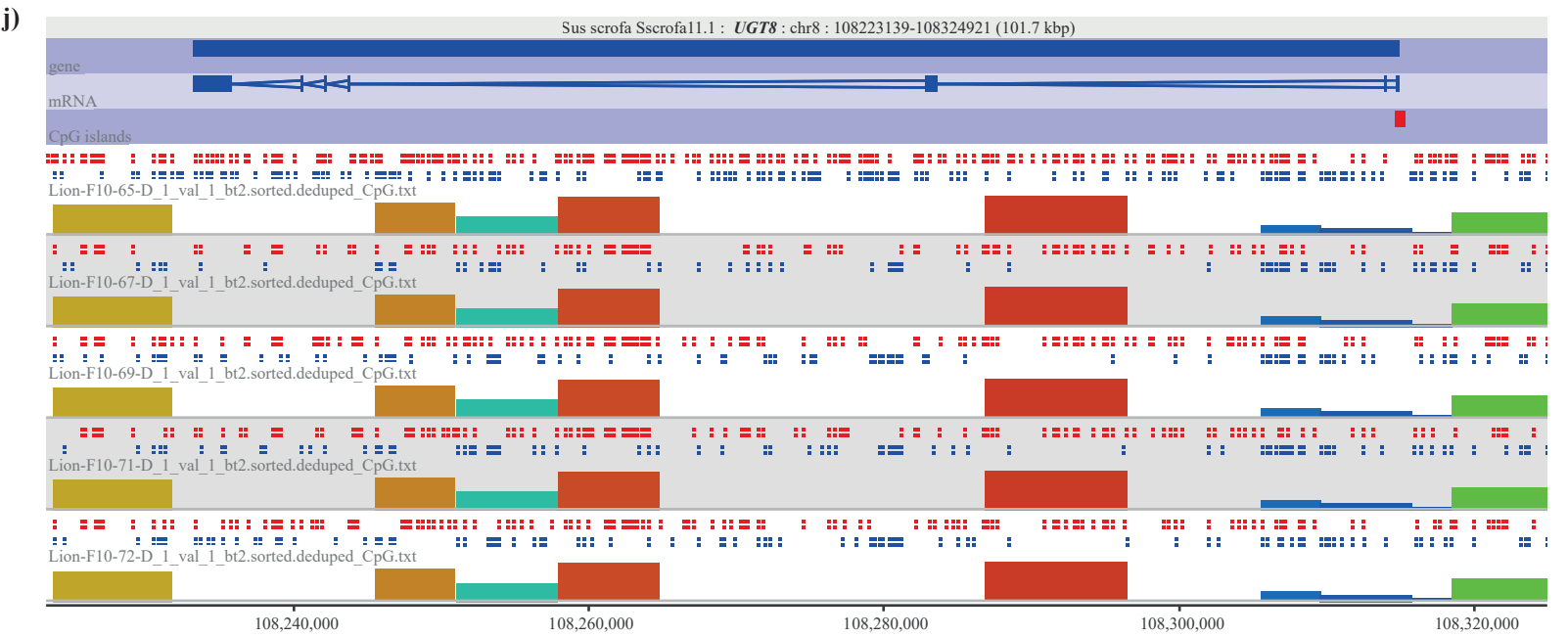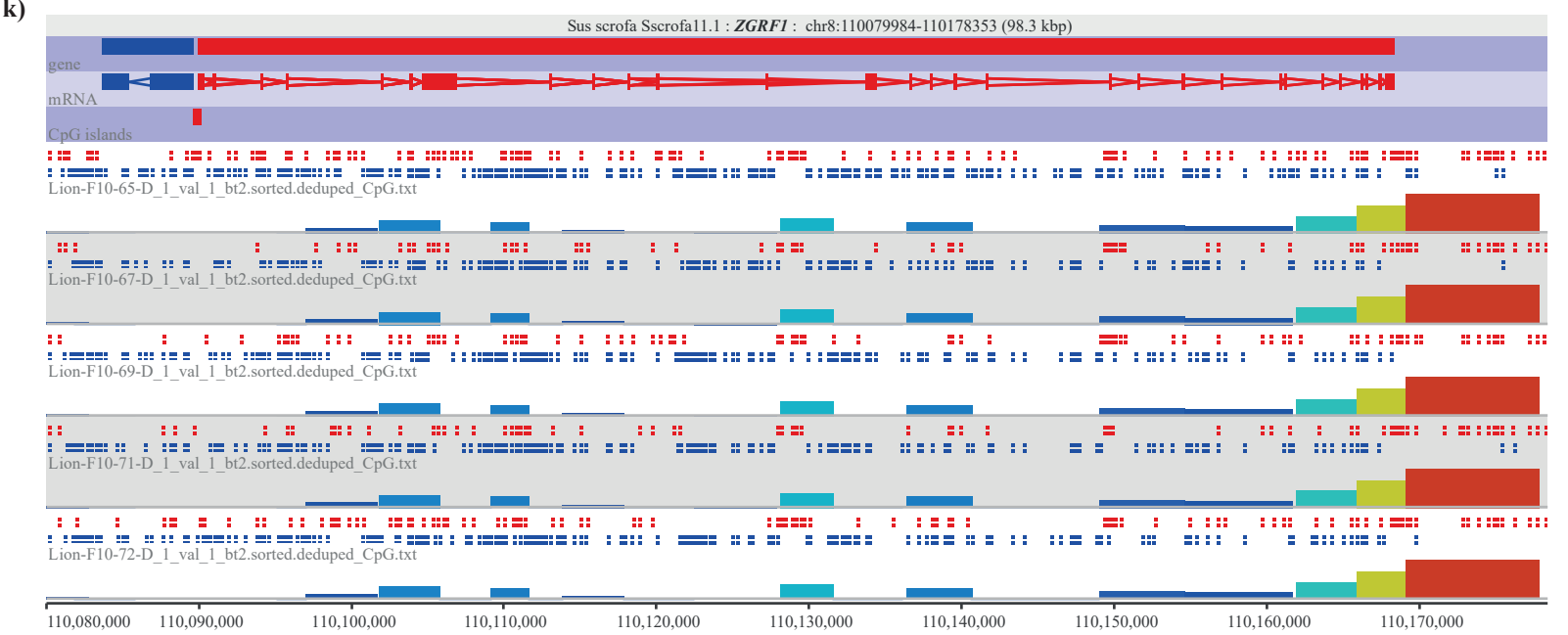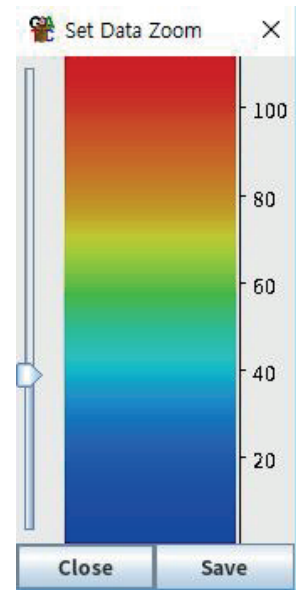

Supplement: Supplementary file 11 — Supplementary Information 11. [file 41598_2021_86683_MOESM11_ESM.pdf]

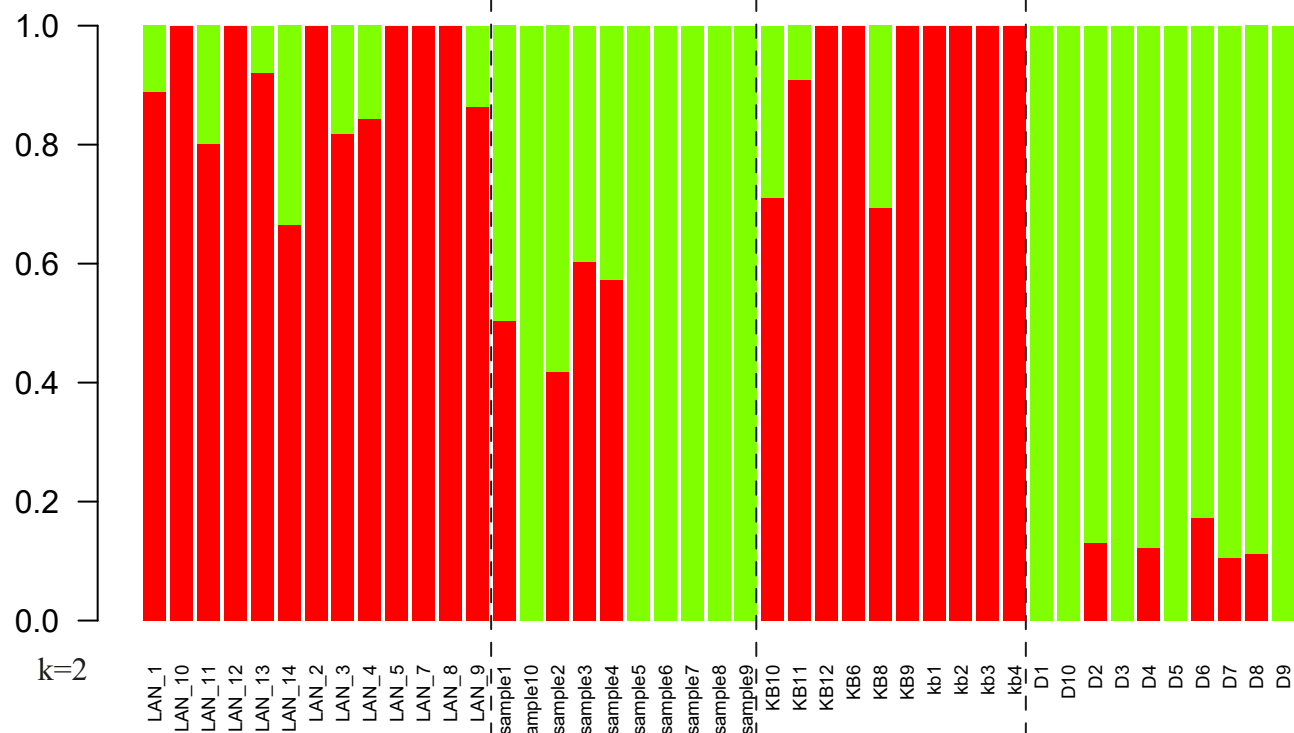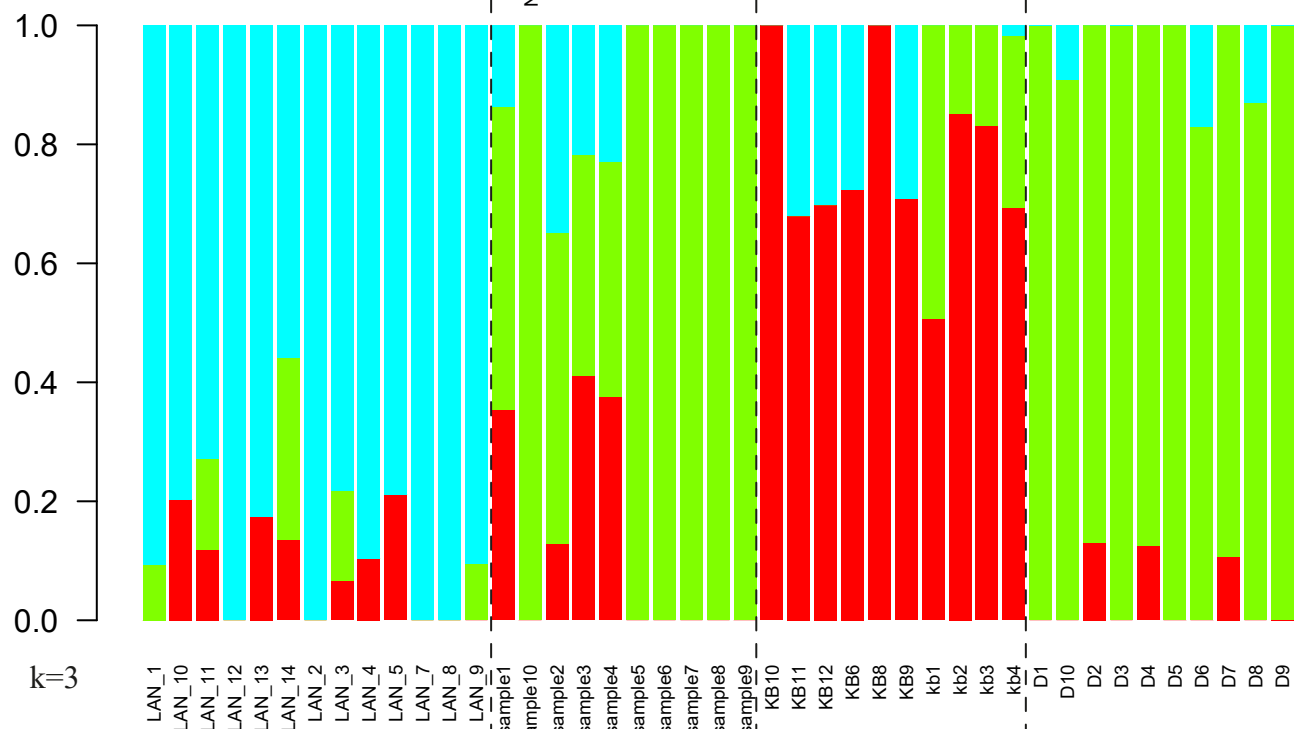

Landrace

Nanchukmacdon

Jeju native pig

Duroc

Supplement: Supplementary file 12 — Supplementary Information 12. [file 41598_2021_86683_MOESM12_ESM.pdf]

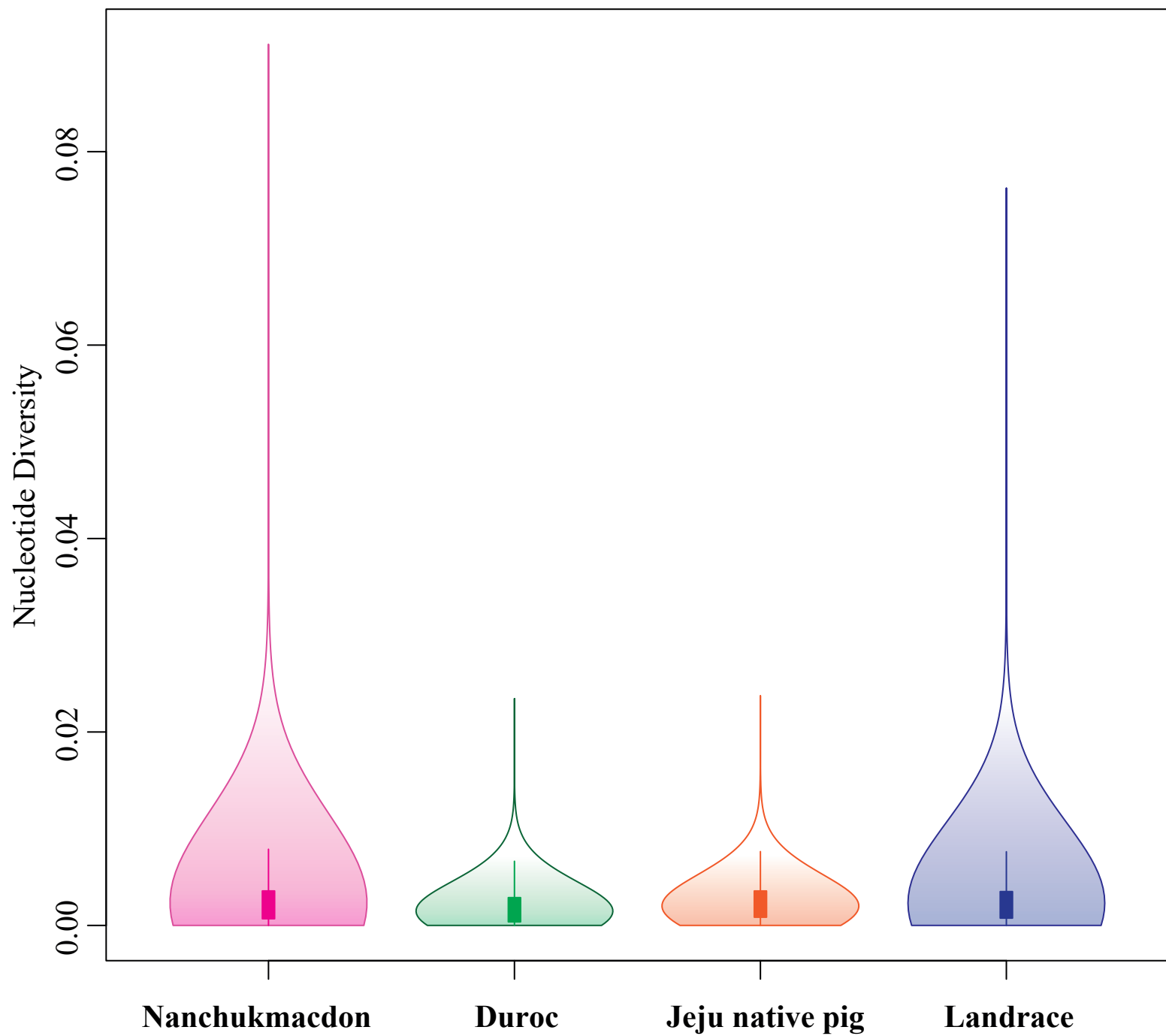

Supplement: Supplementary file 13 — Supplementary Information 13. [file 41598_2021_86683_MOESM13_ESM.pdf]

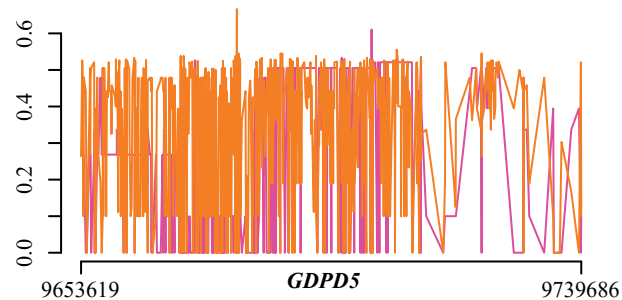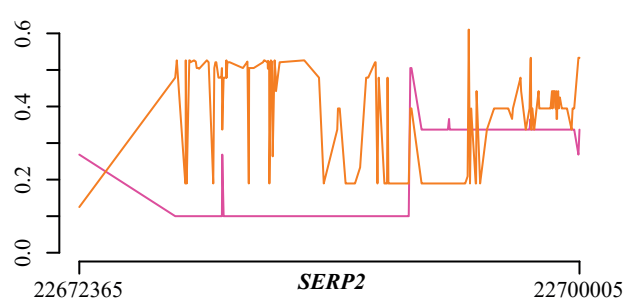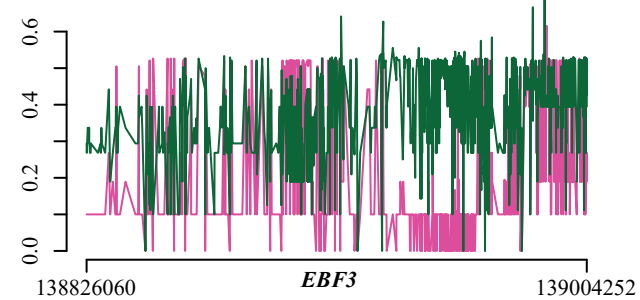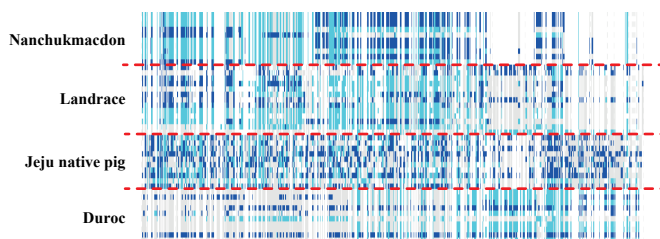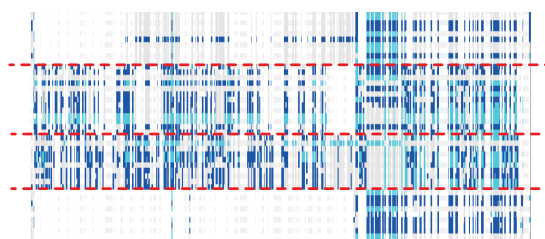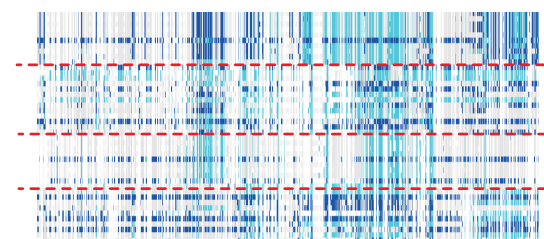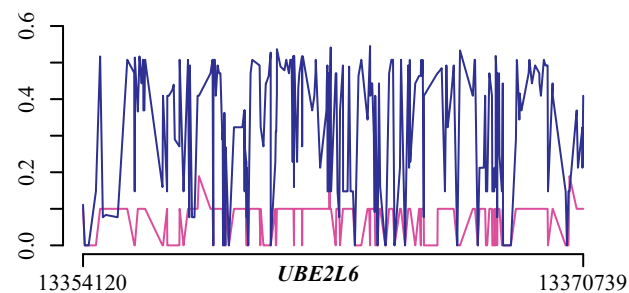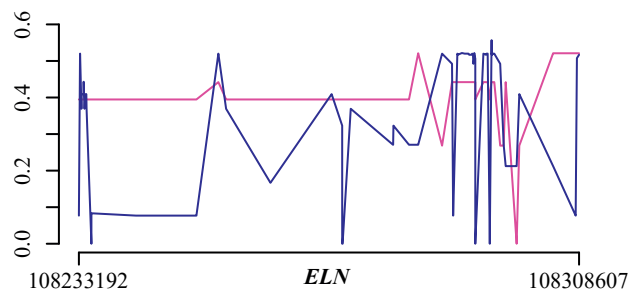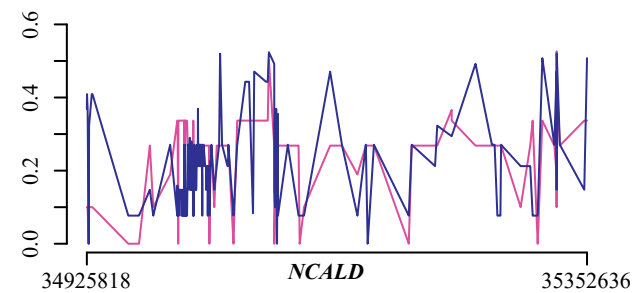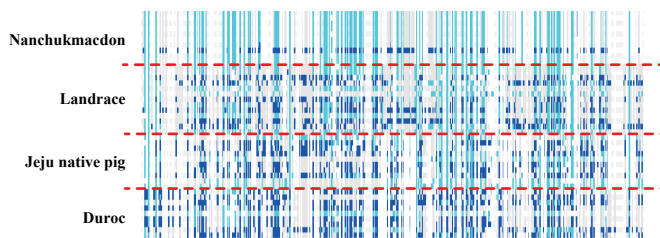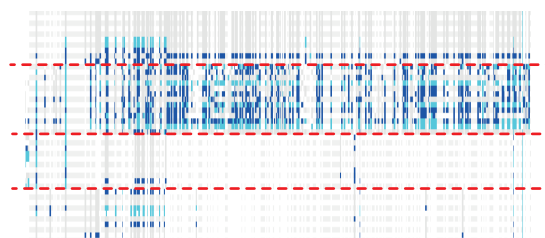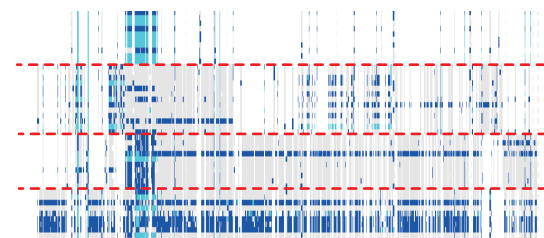

— Nanchukmacdon

— Jeju native pig

— Duroc

— Landrace

Supplement: Supplementary file 14 — Supplementary Information 14. [file 41598_2021_86683_MOESM14_ESM.pdf]
