# Supplementary material for: Integration of multi-omics approaches for functional characterization of muscle related selective sweep genes in Nanchukmacdon
Source: Sci Rep. 2021 Mar 30;11:7219. doi: 10.1038/s41598-021-86683-4 (PMC8009959; doi:10.1038/s41598-021-86683-4)
Supplement: Supplementary file 9 — Supplementary Information 9. [file 41598_2021_86683_MOESM9_ESM.pdf]

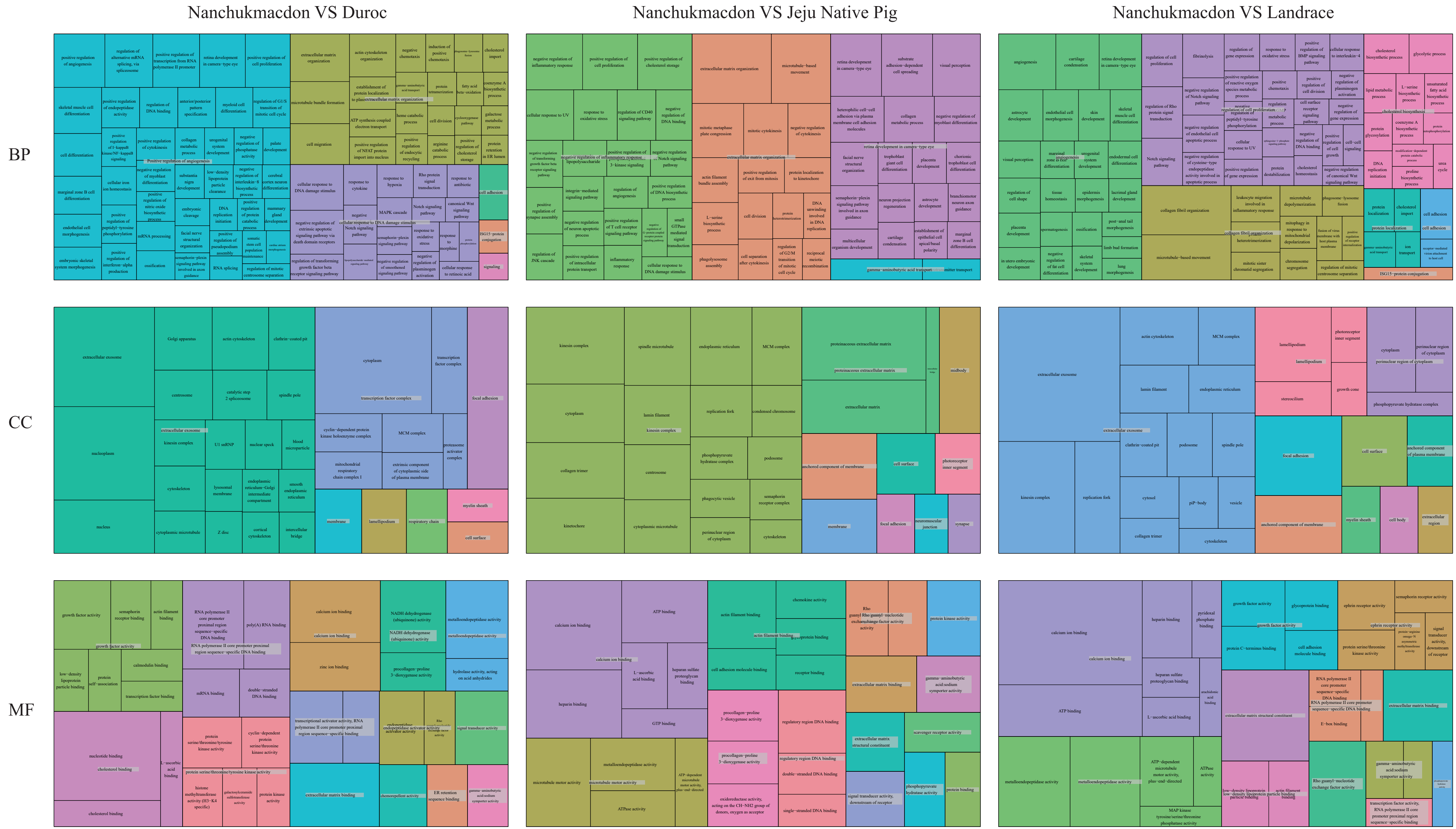
Nanchukmacdon VS Duroc
Nanchukmacdon VS Jeju Native Pig
Nanchukmacdon VS Landrace
BP
CC
MF
