# Supplementary material for: Integration of multi-omics approaches for functional characterization of muscle related selective sweep genes in Nanchukmacdon
Source: Sci Rep. 2021 Mar 30;11:7219. doi: 10.1038/s41598-021-86683-4 (PMC8009959; doi:10.1038/s41598-021-86683-4)
Supplement: Supplementary file 15 — Supplementary Information 15. [file 41598_2021_86683_MOESM15_ESM.docx]

**Supplementary file**

**Integration of multi-omics approaches for functional characterization of muscle related selective sweep genes in Nanchukmacdon**

Devender Arora^1^, Krishnamoorthy Srikanth^1,4^, Jongin Lee^3^, Daehwan Lee^3^, Nayoung Park^3^, Suyeon Wy^3^, Hyeonji Kim^3^, Jong-Eun Park^1^, Han-Ha Chai^1^, Dajeong Lim^1^, In-Cheol Cho^2^, Jaebum Kim^3*^ and Woncheoul Park^1*^

Fig S1a: Summary of gene ontology of Biological process, Cellular compartment and Molecular function involved in selective sweep genes identified from XP-EHH and XP-CLR.

Fig S1b: Summary of gene ontology of Biological process, Cellular compartment and Molecular function involved in DEGs corresponds to Duroc, landrace and Black pig.

Fig S2: KEGG pathway analysis of genes identified from XP-EHH and XP-CLR.

Fig S3: Methylation pattern of each gene visualized in SeqMonk.

Fig S4: Admixture result for population segregation at K2 and K3.

Fig S5: Nuclear Diversity violin plot.

Fig S6: Candidate selective sweep genes in NC. Represent the nuclear diversity at each point of location and their respective haplotype distribution with genomic position biallelic alleles are shown in sky-blue (homozygous variant) and blue (Heterozygous variant).

Additional table 1: Commonly identified selective signature genes with top 1% of XP-CLR and -2 cutoff for XP-EHH score with log2fold change of < 1 and FDR of 0.05 against JNP, Duroc and Landrace.

Additional table 2: GO (BP, CC, and MF) from DEGs in Nanchukmacdon from Duroc, Landrace and BlackPig.

Additional table 3: Primer used for identified gene validation.

Additional Table 4: Phenotypic information of Nanchukmacdon

Additional Data 1: XP-EHH and XP-CLR score for NC from DU, LR and JNP.

Additional Data 2: Degs identified from DSeq2 for NC from DU, LR and JNP.

Additional Data 3: David analysis for genes for NC from DU, LR and JNP in BP, CC, and MF.
